# Chemical Characterization and Cytotoxic and Antioxidant Activity Evaluation of the Ethanol Extract from the Bulbs of *Pancratium maritimun* Collected in Sicily

**DOI:** 10.3390/molecules28103986

**Published:** 2023-05-09

**Authors:** Adele Cicio, Stefania Sut, Stefano Dall’Acqua, Maurizio Bruno, Claudio Luparello, Rosa Serio, Maria Grazia Zizzo

**Affiliations:** 1 Department of Biological, Chemical and Pharmaceutical Sciences and Technologies (STEBICEF), Università degli Studi di Palermo, Viale delle Scienze, ed. 16, 90128 Palermo, Italy; adele.cicio01@unipa.it (A.C.); claudio.luparello@unipa.it (C.L.); rosa.serio@unipa.it (R.S.); mariagrazia.zizzo@unipa.it (M.G.Z.); 2 Department of Pharmaceutical and Pharmacological Sciences, University of Padova, via F. Marzolo 5, 35131 Padova, Italystefano.dallacqua@unipd.it (S.D.)

**Keywords:** Amaryllidaceae, alkaloids, cytotoxic activity, antioxidant activity, Caco-2 cells

## Abstract

*P. maritimum* L., belonging to the Amaryllidaceae family, is a species that grows on beaches and coastal sand dunes mainly on both sides of the Mediterranean Sea and Black Sea, the Middle East, and up to the Caucasus region. It has been largely investigated due to its several interesting biological properties. With the aim of providing new insights into the phytochemistry and pharmacology of this species, the ethanolic extract of the bulbs from a local accession, not previously studied, growing in Sicily (Italy), was investigated. This chemical analysis, performed by mono- and bi-dimensional NMR spectroscopy, as well as LC-DAD-MSn, allowed to identify several alkaloids, three of which were never detected in the genus *Pancratium.* Furthermore, the cytotoxicity of the preparation was assessed in differentiated human Caco-2 intestinal cells by trypan blue exclusion assay, and its antioxidant potential was evaluated using the DCFH-DA radical scavenging method. The results obtained demonstrate that *P. maritimum* bulbs’ extract exerts no cytotoxic effect and is able to remove free radicals at all the concentrations tested.

## 1. Introduction

The family of Amaryllidaceae includes about sixty genera with more than 850 species, and it is largely spread all over the world, with South America and South Africa being the regions with major diversity [1], whereas in the Mediterranean region, only eight genera are present.

Among all the genera of Amaryllidaceae, *Pancratium* is the most widespread [2], distributed in Africa, South Europe, the Middle East, the Arabian Peninsula, and the Indian area [3]. The genus takes its name from the Greek word “pagkration”, meaning almighty, due to the numerous medicinal properties of some species, and, according to POWO [3], twenty-four taxa have the rank of accepted species.

**Figure 1:**
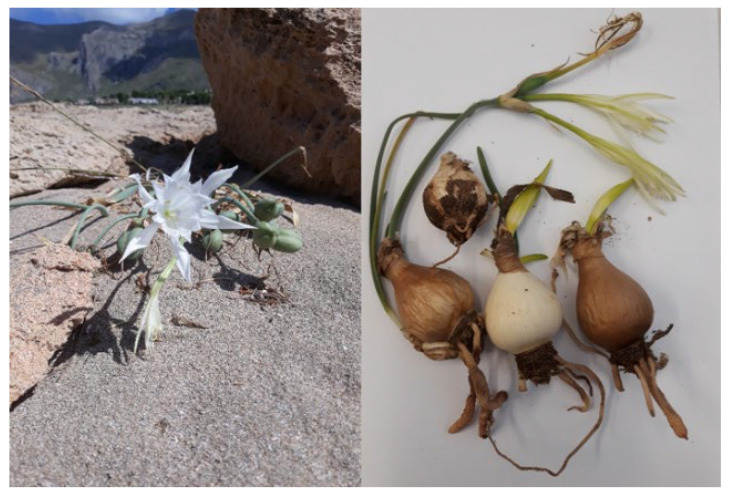
*P.maritimum* L. (**left**) and its bulbs (**right**).

*Pancratium maritimum* L. (marine narcissus, sea daffodil) (Figure 1) is a bulbous perennial plant with a long neck and glaucous, broadly linear leaves, evergreen, but the leaves often die back during hot summers. The white flowers (3–15 in an umbel) are long, up to 15 cm, with a corona two-thirds as long as the tepals. It flowers from August up to October [4,5].

It mainly grows on beaches and coastal sand dunes, often with some leaves in the sand, and it is native to both sides of the Mediterranean Sea and Black Sea, the Middle East, and up to the Caucasus region [3]. However, recently, due to urbanization, tourism development, alteration, and destruction of dune systems, its populations have drastically declined [6].

The first phytochemical study on this plant dates from 1954 [7] and is concerned with the identification of some alkaloids. Since then, several other studies have shown the occurrence of many alkaloids belonging to the lycorine-, lycorenine-, montaninetazettine-, galanthamine-, haemanthidine-, crinine-, isocarbostiryl-, narciclasine-, and pancratistatin groups, all biogenetically formed from norbelladines [8]. Alkaloids with these skeletons have been identified in plants, mainly bulbs, collected in Egypt [9,10,11,12,13,14,15,16], Bulgaria [17], Turkey [18,19,20,21,22], Tunisia [23], Portugal [24], Spain [25,26], and Italy (Calabria) [27]. The occurrence of the alkaloids in the bulbs of the different accessions is reported in Table 1.

Apart from alkaloids, other metabolites have been identified in the bulbs or aerial parts of *Pancratium maritimum* L. (*P. maritimum)* such as chromones, [30,31,32], flavonoids [30,31,33], phenylpropanoids [16], phenolic acids [33,34], and alginates [23,35].

Furthermore, the extracts of different accessions of *P. maritimum,* as well as the pure isolated compounds, have been investigated for their cytotoxic [14,16,24,27,36,37,38,39], antimicrobial [14,15,40,41], antiviral [27], antioxidant [34,38], antinociceptive [42], amoebicidal [43], antimalarial [22], and acetylcholinesterase inhibitory properties [18,44,45].

In the frame of the ongoing research on Mediterranean plants with antioxidant properties [46,47,48,49,50], we describe here for the first time the chemical composition of a not previously studied accession of *P. maritimum* growing wild in Sicily. HPLC-DAD analysis is frequently applied for the detection of such compounds in Amarillidaceae and allows the separation of different derivatives and their quantification. LC-MS and GC-MS have also been used [51] to detect such constituents in different plant extracts, offering the opportunity for their quali/quantitative analysis also in low concentrations. In this work, a simple HPLC-DAD-MS method using reverse-phase chromatography was used. In addition, its cytotoxic and antioxidant activities were evaluated on differentiated Caco-2 cells. Caco-2 cell cultures, even if cancerous in origin, undergo a gradual differentiation process in enterocytes that takes place spontaneously once confluence has been reached. Thus, they represent an established “in vitro” test system for the evaluation of the biological activity of natural compounds [52].

## 2. Results and Discussion

### 2.1. Chemical Characterization

#### 2.1.1. Nuclear Magnetic Resonance Analysis of Bulbs’ Extract from *P. maritimum*

**Figure 2:**
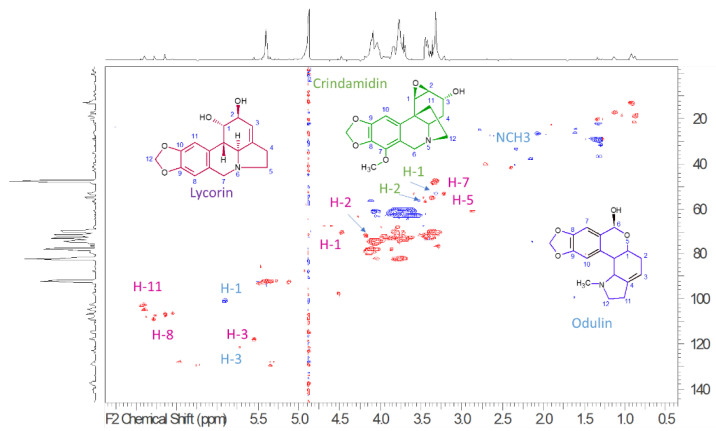
HSQC-DEPT of ethanol bulbs’ extract of *P. maritimum*, blue peaks are representing the CH_2_, while red peaks are the CH and CH_3_. In the spectrum the diagnostic positions assigned to the most abundant alkaloid derivatives are reported in different colour. Lycorin diagnostic signals are highlighted in purple color, crindamidin in green color, and odulin in blue color.

To establish the presence of main constituents, ^1^H-NMR, HSQC-DEPT, HMBC, COSY, TOCSY, and NOESY spectra (Supplementary Materials) were acquired and allowed to recognize the presence of different constituents. ^1^H-NMR spectra (Supplementary Materials) showed a crowded region due to carbohydrate constituents, and, due to the presence of broad peaks, at least part of these compounds can be related to the presence of oligo or polysaccharides. Several signals can be diagnostic of Amaryllidaceae alkaloids, namely a series of N-linked CH presenting δ_H_ 3.20–3.60 and carbon resonances at δ_C_ 52.0–58.0. Furthermore, aromatic signals and singlets support the presence of the methylenedioxy group. In particular, some diagnostic signals can be ascribed to the presence of lycorin-type alkaloids and are highlighted in the Figure 2 that present the HSQC-DEPT spectrum of the extract. Main signals are the N-linked CH_2_ assigned to the positions C-4 (δ_H_ 2.42; δ_C_ 33.72), C-5 (δ_H_ 3.32; δ_C_ 52.97), the CHOH C-1 at (δ_H_ 4.57; δ_C_ 7.051), C-2 (δ_H_ 4.27; δ_C_ 71.8), and the sp^2^ CH in position C-3 (δ_H_ 5.64; δ_C_ 117.3). H-2, H-3 coupling is observed in the COSY spectrum; furthermore, in the TOCSY spectrum, we can observe cross peaks that allow to establish connections between H-5, H-4, H-3, and H-2. Other diagnostic signals can be related to the aromatic part of the compound, namely the C-8 (δ_H_ 6.74; δ_C_ 107.8), C-11 (δ_H_ 6.98; δ_C_ 104.8), and the methylene dioxy C-12 (δ_H_ 6.02; δ_C_ 101.07) that are part of one aromatic ring, as demonstrated by the HMBC correlation observed from the H-12 and from the H-11 and H-8 to the oxygenated carbons C-9 and C-10. The obtained information suggests the presence of the pyrrolo [d,e] phenanthridine ring system and is in agreement with literature data for lycorine-type alkaloids [53,54,55,56]. Signals also suggest the presence of crinamidine-type alkaloids [53,54,55,56], namely due to the observation of CH linked to epoxy groups ascribable to C-1 (δ_H_ 3.77; δ_C_ 54.3) and C-2 (δ_H_ 3.26; δ_C_ 53.3) and the latter coupling with CHOH at C-3 (δ_H_ 4.46; δ_C_ 70.8). Furthermore, N-linked CH_2_ C-6 (δ_H_ 4.19–3.53; δ_C_ 56.4) and C-12 (δ_H_ 3.31–2.43; δ_C_ 53.1) can be observed. The presence of lycorenine or oduline-type alkaloids can be suggested due to the resonance observed at δ_H_ 5.93; δ_C_ 97.10 that appears as a singlet ascribable to position C-6 that is emiacetalic, and the C-3 observed at δ_H_ 5.53; δ_C_ 117.3 being in good agreement with the assignment reported from Codina et al. [57].

#### 2.1.2. LC-DAD-MS^n^ Analysis of Bulbs’ Extract from *P. maritimum*

**Figure 3:**
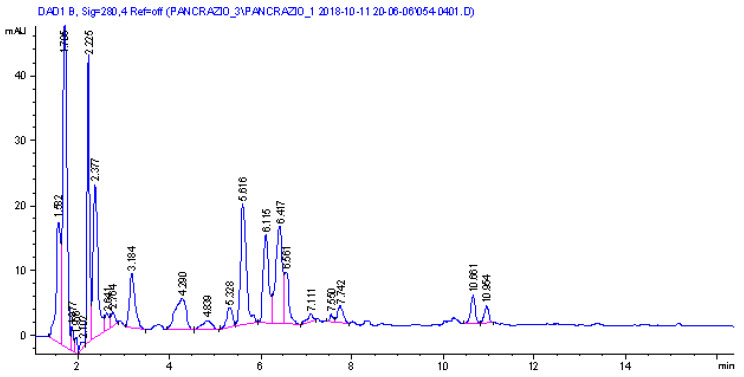
LC-DAD of the methanol bulbs extract of *P. maritimum*.

The DAD trace is reported in Figure 3 and revealed the presence of seven main peaks that present a UV/visible spectrum compatible with lycorine, oduline, and crindamine-type alkaloids. In order to obtain structural information on the eluted compounds, mass spectrometry in positive ion mode using an electrospray ion source was used. An ion trap was the analyzer, and multiple-stage fragmentation mass spectrometry was used to generate fragmentation schemes for each eluted species. The experimental data (see Supplementary Materials) were compared with the literature [58,59,60,61,62,63], allowing tentative identification of the compounds.

Main ions ascribable to alkaloids were observed at *m*/*z* 334, 288, 302, 318, and 306. Two main peaks presenting *m*/*z* 334 were observed at 1.8 and 2.3 min, and their fragmentation allows for a tentative assignment to 2-hydroxy-3-dihydro-6-*O*-methyl-oduline and one isomer. Four peaks presenting [M + H]^+^ at *m*/*z* 288 and a similar fragmentation pattern were assigned to lycorine isomers. Due to the presence of the two different OH groups in Lycorine, the four different peaks with identical MS/MS fragmentation suggest the occurrence in the sample of the four isomers due to the orientation of positions 1 and 2. Six different peaks present [M + H]^+^ at *m*/*z* 302, and we can observe two different schemes of fragmentation ascribable to oduline for the peaks with retention times at minutes 3.33, 4.08, and 4.40, while tentatively assigned to methoxy lycorine for the peaks at retention times 6.05, 7.38, and 7.84 min. More in detail, the oduline diagnostic fragments were the ions at *m*/*z* 284, 266, 255, while for the methoxy lycorine, the ions were at *m*/*z* 270, 266, 252, 227, and 182. One relevant peak was observed at 5.6 min, presenting [M + H]^+^ at *m*/*z* 318, and it was assigned to crinamidine. Two peaks were observed presenting [M + H]^+^ at *m*/*z* 306 and were assigned to crinamabine and its isomer.

For quantitative purposes, galanthamine was used as a reference compound due to its commercial availability and relative similarity in structure. Galanthamine was not detectable in the analyzed extract. The amount of alkaloids detected in the bulbs’ extract is reported in Table 2.

**Figure 4:**
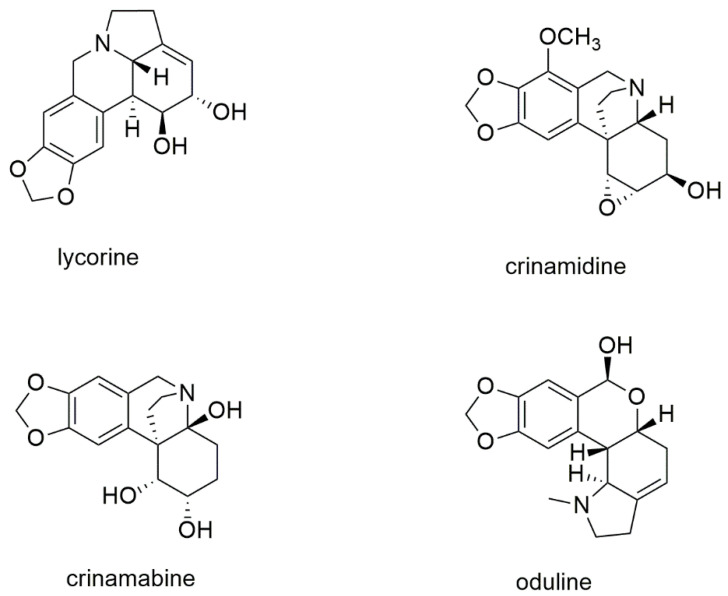
Structure of the most abundant alkaloids in the bulbs’ extract of *P. maritimum*.

The structure of the most abundant organic constituents of the bulbs’ extract of *P. maritimum* is reported in Figure 4.

The main constituent of the extract was lycorine, an alkaloid found practically in all the other accessions of *P. maritimun* studied so far (Table 1). On the other hand, it is very interesting to point out that the other three main alkaloids, crinamidine, crinamabine, and oduline, have never been detected in any other extract of *P. maritimun* previously investigated, nor in any other species of the genus *Pancratium*.

Crinamabine was first isolated from the bulbs of Crinum amabile Donn. (Amaryllidaceae), collected in Vietnam [64], and successively identified only in the leaves of Crinum asiaticum L. var. sinicum from Taipei [65]. Oduline has been identified in the bulbs of Narcissus pseudonarcissus subsp. pseudonarcissus cv. Carlton (Amaryllidaceae) [66] and Narcissus poeticus cv. Golden Ducat [67], from plants cultivated in Holland and the Czech Republic, respectively, and in the bulbs of two Chinese species, *Lycoris aurea* Herb. (Amaryllidaceae) [68] and *Lycoris radiata* Herb. [69]. The distribution of crinamidine is quite large. In fact, it has been detected in the bulbs of several Amaryllidaceae species such as *Crinum bulbispermum* Milne-Redhead et Schweickerdt [70], *C. latifolium* (L.) [71], *C. macowanii* Baker [72], *Nerine bowdenii* W. Wats [73,74], *N. flexuasa* Herb. [75], *Brunsvigia gregaria* R. A. [76], *B. orientalis* (L.) Ait ex Eckl [77], *Ammocharis tinneana* (Kotschy & Peyr.) Milne-Redh. & Schweick [78], and *Zephyranthes grandiflora* [79], as well as, recently, in the leaves of *Carica papaya* L. [80], belonging to the Caricaceae family.

### 2.2. Absence of Cytotoxic Effect by P. maritimum Bulbs’ Extract on Human Intestinal Cells

**Figure 5:**
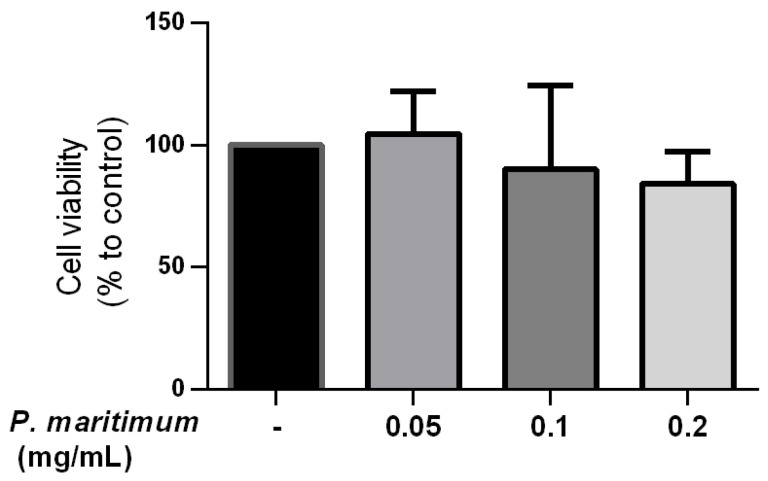
Effect of *P. maritimum* bulbs’ extract on cell viability in differentiated Caco-2. The cytotoxic effects of exposure to *P. maritimum* bulbs’ extract (0.05–0.2 mg/mL) for 24 h were assessed by trypan blue assay. The data were represented as means ± SEM of three independent experiments.

**Figure 6:**
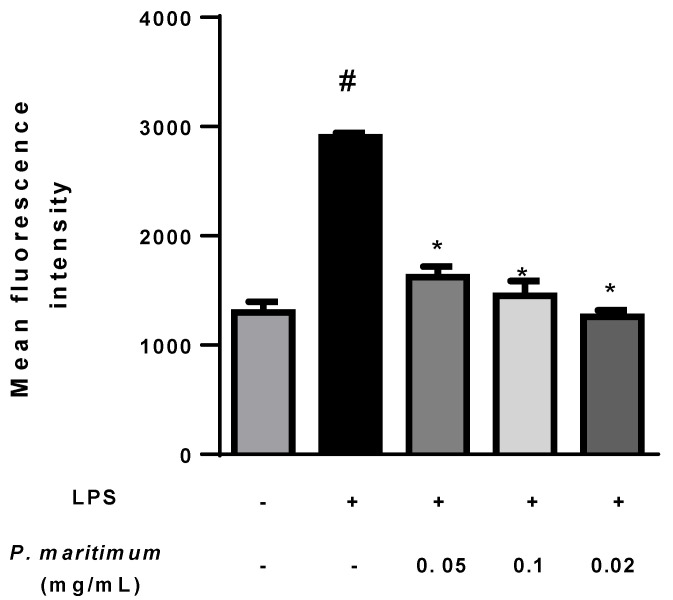
Effect of *P. maritimum* bulbs’ extract on intracellular reactive oxygen species (ROS) production in lipopolysaccharide (LPS)-stimulated differentiated Caco-2 cells. Intracellular ROS was analyzed using fluorescent probe H_2_DCF-DA in cells untreated (negative control (−)), treated with only 0.1 mg/mL LPS ((+) positive control), or co-treated with *P. maritimum* bulbs’ extract (0.05–0.2 mg/mL) and with LPS (0.1 mg/mL) for 24 h. The ROS production was determined as mean fluorescence intensity of dichlorofluorescein (DCF). The data were represented as means ± SEM of three independent experiments. Confidence intervals calculated by one-way ANOVA and Tukey post hoc test: # significant vs. untreated control cells: *p* < 0.001; * significant vs. LPS-treated cells: *p* < 0.001.

The first step in evaluating the suitability of plant extracts for further applications is the assessment of their potential cytotoxicity (Figure 5). In our study, the trypan blue exclusion method on Caco-2 differentiated cells was used. Cells were treated for 24 h with concentrations of *P. maritimum* bulbs’ extract ranging from 0.05 to 0.2 mg/mL. As shown in Figure 6, no significant effect on cell viability was observed at all the concentrations tested. In particular, the maximal decrease in cell viability observed, down to 84% compared to controls, was detected at a concentration of 0.2 mg/mL. Although cytotoxic effects of *P. maritimum* and its alkaloid components have been reported in different tumor cell lines [16,39,81,82], our data are in agreement with the observations indicating the absence of anti-proliferative and death-inducing effects in normal cell culture, as in human lymphocytes [39].

### 2.3. Down-Regulation of Reactive Oxygen Species (ROS) Production in LPS-Induced Cells by P. maritimum Bulbs’ Extract

Many studies have shown that ROS production, which leads to oxidative stress, plays a major role in the hyperactivation of inflammatory processes [83].

The inflammatory mediators, such as cytokines, accelerate the accumulation of intracellular ROS that, as secondary messengers, would participate in the inflammatory signaling pathway [84].

Several studies report the antioxidant potential of *P. maritimum* stems, flowers, and bulbs [38,44,85]. These effects appear to be related to the high contents of phenols and flavonoids [33] and also to the lycorine alkaloid [86].

The effect of our bulb extracts on intracellular ROS levels was investigated using a cell-based assay in which H_2_DCF-DA, a fluorescent probe, is used as an indicator of ROS and oxidative stress. The H_2_DCF-DA probe passively diffuses into the cells and is hydrolyzed by intracellular esterases to form the non-fluorescent 2′,7′-dichlorofluorescein (H_2_DCF). In the presence of ROS, intracellular oxidases, and oxidants, H_2_DCF is oxidized to fluorescent 2′,7′-dichlorofluorescein (DCF), remaining in the cells [87]. Antioxidants would prevent the generation of DCF.

To induce inflammation, Caco-2 cells were stimulated with lipopolysaccharide (LPS) in order to produce pro-inflammatory cytokines and other inflammatory mediators, including ROS [88]. As expected, we observed in differentiated Caco-2 cells exposed to 0.1 mg/mL of LPS for 24 h an enhanced ROS production of about 130% compared to the control (Figure 6).

The treatment with increasing concentrations of *P. maritimum* bulbs’ extract down-regulated ROS production compared to the positive control, suggesting an antioxidant effect.

We can conceivably suppose a pivotal role in the down-regulation of ROS production played by alkaloids such as lycorine and lycorine-like compounds present in the composition of Sicilian *P. maritimum* bulbs’ extract. However, further studies are needed to solve this issue. Moreover, it will be interesting to investigate whether the inhibition of ROS generation induced by *P. maritimum* extract may have the potential to inhibit the expression of pro-inflammatory mediators and cytokines.

## 3. Materials and Methods

### 3.1. Plant Material

Bulbs of *P. maritimum* L. (see Figure 1) were collected from plants at their full flowering stage in September 2021 at Salinelle (38°01′48″ N, 13°18′40″ E, 16 m s/l), Lascari (Palermo), in Sicily, Italy, on a sandy soil. Typical specimens, identified by Mr. Emanuele Schimmenti, have been deposited in the Department STEBICEF, University of Palermo, Palermo, Italy (voucher No. MB 385/21).

### 3.2. Extraction of Plant Material

The ethanolic extract of *P. maritimum* was obtained from bulbs according to the following procedure. After washing and air-drying, the bulbs’ parts were pulverized by using an electric blender. About 100 g of the dried sample was soaked in 99% ethanol in a 1000 mL volumetric flask. The flask was covered and allowed to stand for 48 h at room temperature. After filtration, the excess solvent was removed by using a rotary evaporator. The dried residue (7.23% yield) was stored in an air-sealed analytical container at 4 °C.

### 3.3. Chemical Analysis

Bulbs’ extract was dissolved in deuterated methanol (10 mg/mL) and used for the ^1^H NMR and 2D NMR (HSQC, HMBC, and COSY) experiments in order to assess the presence of the main constituents.

Heteronuclear correlation spectroscopy gives signals based on coupling between nuclei of two different types. Heteronuclear single-quantum correlation spectroscopy (HSQC) detects correlations between nuclei of two different types that are separated by one bond. Heteronuclear multiple-bond correlation spectroscopy (HMBC) detects heteronuclear correlations over longer ranges of about 2–4 bonds. The homonuclear correlation spectroscopy (COSY) sequence is used to identify spins that are coupled to each other.

NMR data were considered a preliminary investigation, and then, in order to assess the presence of the compounds in the extract and also to perform quantitative analysis, the extract was subjected to LC-DAD-MS/MS in an ion trap in positive ion mode.

Qualitative characterization and quantitative analysis of metabolites from bulbs’ extract of *P. maritimum* were achieved by liquid chromatography hyphenated with photodiode-array detection and tandem electrospray ionization mass spectrometry (LC-DAD-ESI-MS^n^) in an ion trap in positive ion mode. The extract of the bulbs was dissolved in methanol and sonicated, obtaining a solution at 5 mg/mL. After centrifugation, the solution was used for analysis by means of an Agilent 1260 chromatograph equipped with a diode array (DAD) 1260 series and an MS 500 ion trap mass spectrometer. Spectra were acquired in positive ion mode in the range of *m*/*z* 50–1000. Fragmentation of the main ionic species was obtained using the turbo data-dependent scan (tdds) function of the instrument. UV spectra were obtained in the DAD detector in the range of 200–400 nm, and galanthamine chloridrate was used as a reference compound and also as a reference for the semiquantification of alkaloids. A stock solution of galantamine chloridrate was prepared in methanol at a concentration of 50 µg/mL, diluted to 20 µg/mL, 10 µg/mL, 5 µg/mL, and 1 µg/mL, and used to perform a calibration curve.

### 3.4. Cell Culture, Differentiation and Treatments

The Caco-2 cells, taken from laboratory stocks, were cultured in a high-glucose Dulbecco’s Modified Eagle’s Medium (DMEM) (Sigma-Aldrich, Inc., St. Louis, MO, USA) supplemented with 10% fetal bovine serum (Life Technologies, Carlsbad, CA, USA) and antibiotics (100 U/mL penicillin and 100 μg/mL streptomycin) (Sigma-Aldrich, Inc., St. Louis, MO, USA) at 37 °C in a humidified 5% CO_2_ incubator. For the experimental studies, 3.5 × 10^4^ cells/cm^2^ were grown in 24-well plates and used after 21 days, when fully differentiated. For this purpose, once cells reached 100% confluence four days after seeding, the medium was changed every other day. The dome formation, as a hallmark of the differentiated phenotype, was observed under the microscope [89].

The dry extract was dissolved in DMSO and added to the cell medium at concentrations ranging from 0.05 to 0.2 mg/mL, as previously reported [38,44].

### 3.5. Trypan Blue Exclusion Assay

Cell viability was assessed using the trypan blue exclusion assay [90]. Briefly, the differentiated Caco-2 cells were treated with different concentrations of the extract (0.05–0.2 mg/mL) and DMSO vehicle for 24 h, trypsinized with a 1:30 dilution of standard trypsin-EDTA solution, resuspended in DMEM, and stained with a 0.4% trypan blue dye solution. The number of unstained viable cells was quantitated in a Burker hemocytometer under a phase contrast microscope. The percentage of cell viability was calculated as the number of treated viable cells/number of control viable cells × 100. Each experiment was performed in triplicate.

### 3.6. Assay for Cellular Antioxidant Activity

The intracellular accumulation of ROS was evaluated using the ROS Detection Assay Kit (Canvax Biotech, Cordoba, Spain), which uses the cell-permeant reagent dichlorodihydrofluorescein-diacetate (H_2_DCF-DA), a fluorogenic dye that measures hydroxyl, peroxyl, and other ROS activity within the cell. After diffusion into the cell, the acetyl groups on H_2_DCF-DA are cleaved by intracellular esterase to yield the non-fluorescent compound, which is rapidly oxidized to highly fluorescent 2′,7′-dichlorodihydrofluorescein by ROS. The fluorescence intensity is proportional to the ROS levels within the cell cytosol.

The Caco-2 cells, differentiated in 24-well cell culture plates, were exposed to 0.1 mg/mL LPS supplemented with the extract at concentrations of 0.05, 0.1, and 0.2 mg/mL. The negative control (DMSO vehicle only) and the positive control (0.1 mg/mL LPS only) were included in the assay.

ROS production was analyzed by flow cytometry [91]. Three assays were performed on treated and control cells using a FACSCanto instrument (BD Biosciences, Franklin Lakes, NJ, USA) in the FL1 channel (Ex/Em = 485/530). Ten thousand events were assessed, and the obtained data were analyzed with the Floreada analysis tool available at https://floreada.io (accessed on 10 December 2022). Results are expressed as the mean intensity fluorescence of DCF (λ = 495 nm).

### 3.7. Statistical Analyses

Data are reported as the mean ± SEM. A one-way analysis of variance (ANOVA) followed by Tukey’s test was performed in order to highlight possible significant differences between groups (*p* < 0.001) using the GraphPad Prism 6 software (GraphPad Software, San Diego, CA, USA).

## 4. Conclusions

In conclusion, our findings suggest that Sicilian *P. maritimum* L. is a good source of Amaryllidaceae alkaloids, namely lycorine, oduline, and crindamine. The obtained data revealed that this sample presents a peculiar alkaloid profile with respect to the other accessions studied so far. This suggests the need for further studies on Sicilian *P. maritimum* L. to deeply investigate the chemical diversity of this plant material that can become a valuable source of bioactive constituents. Furthermore, the obtained data indicate that this plant can supply antioxidant compounds as potent radical scavengers by inhibiting ROS production while conversely exerting no cytotoxic activity on differentiated Caco-2 cells as a model of the gut epithelium at the range of concentrations tested under the experimental conditions used in the present work.

## Figures and Tables

**Table 1:** Alkaloids in the bulbs of other accessions of *Pancratium maritimum* L. (*P. maritimum*).

Origin	Alkaloids	Ref
Bulgaria	Trispheridine, graciline, galanthamine, buphanisine, *N*-demethylgalanthamine, crinine, demethylmarithidine, haemanthamine, tazettine, pancracine, lycorine	[17]
Caucasus	Tazettine, lycorine	[28]
Egypt	Narciclasine-4-*O*-β-glucopyranoside	[9]
Egypt	Ungeremine, zefbetaine, lycorine, tazettine, pancracine, lycorenine, galanthamine, sickenbergine, homolycorine, haemanthidine, hippadine, demethylhomolycorine, trispheridine, haemanthamine, pseudolycorine, 9-*O*-demethylhomolycorine, 11-hydroxyvittatine	[10]
Egypt	Ungeremine	[15]
Egypt	Pancrimatine A, pancrimatin B	[11]
Israel	Pancratistatin	[29]
Italy, Calabria	Lycorine, 9-*O*-demethyllycorine, haemanthidine, haemanthamine, 11-hydroxyvittatine, homolycorine, pancracine, obliquine, tazettine, vittatine.	[27]
Portugal	Haemanthidine, hippeastrine, lycorine, 11α-hydroxygalanthamine, 2α-10bα-dihydroxy-9-*O*-demethylhomolycorine, *epi*-galanthamine, 8-*O*-demethylhomolycorine, tazettine, haemanthamine	[24]
Spain	Ungiminorine *N*-oxide	[25]
Spain	6-*O*-methylhaemanthidine, *O*,*N*-dimethylnorbelladine, lycorine, hippeastrine, galanthamine, haemanthamine, vittatine, 11-hidroxyvittatine, hordenine, 9-*O*-demethylhomolycorine, habranthine, ungiminorine, ungiminorine *N*-oxide	[26]
Tunisia	Narciclasine, lycorine, crinine (a), galanthamine, crinine (b), tazettine	[23]
Turkey	Lycorine, galanthamine, crinine, pancracine, 1-acetyl-*β*-carboline, 11,12-dehydrolycorene, galanthindole, 2,11-didehydro-2-dehydroxylycorine, assoanine, *O*-methylpretazettine, hordenine, 1-acetyl-*β*-carboline, 11,12-dehidrolycorene, buphanasine, 2,11-didehydro-2-dehydroxy-lycorine	[18]
Turkey	(−)-3*β*, 11*α*-dihroxy-1,2-dehydrocrinane, (−)-8-hydroxy-9-methoxycrinine	[19]
Turkey	Lycorine, (+)-demethylhomolycorine	[20]
Turkey	(−)-*N*-demethyl-galanthamine, (+)-tazetine, 2-*O*-demethylmontanine	[21]

**Table 2:** LC-DAD-ESI-MS^n^ data and putative identification and semiquantitative amount % of metabolites in the bulbs’ extract of *P. maritimum*.

t_R_ (min)	M + H	Fragments	Identification	% ^a^
1.8	334	316 > 298–280 (265–221–249–237)	2-hydroxy-3-dihydro-6-*O*-methyl-oduline	Traces
1.9	288	270 > 252–222–237–178–149	Lycorine	3.18 ± 0.05
2.34	334	316 > 298–280 (265–221–249–237)	Isomer of the 2-hydroxy-3-dihydro-6-*O*-methyl-oduline	Traces
2.4	288	270 > 252–222–237–178–149	*epi*-Lycorine	1.16 ± 0.02
3.33	302	284 266 255 193	Oduline	0.31 ± 0.02
3.9	288	270 > 252–222–237–178–149	*epi*-Lycorine isomer 1	Traces
4.08	302	284 266 255 193	Oduline isomer 1	Traces
4.4	288	270 > 252–222–237–178–149	*epi*-Lycorine isomer 2	Traces
4.4	302	284 266 255 193	Oduline isomer 2	Traces
4.85	494	462 492 360 314 300 227 211	Unknown	-
5.00	306	288 270 222 189	*epi*-Crinamabine	0.19 ± 0.01
5.6	318	300 286 268 250 227	Crinamidine	1.20 ± 0.03
6.05	302	270 266 252 227 211 182	Methoxy lycorine	1.86 ± 0.06
7.05	306	288 270 222 189	Crinamabine	0.96 ± 0.01
7.38	302	270 266 252 227 211 182	Methoxyl lycorine isomer 1	Traces
7.84	302	270 266 252 227 211 182	Methoxyl lycorine isomer 2	Traces
			Total-	10.3

^a^ Semiquantitative amount % (expressed as galanthamine on the basis of dried extract).

## Data Availability

Data will be made available from the corresponding author on reasonable request. are available in section “MDPI Research Data Policies” at https://www.mdpi.com/ethics.

## Supplementary Materials

The following supporting information can be downloaded at: https://www.mdpi.com/article/10.3390/molecules28103986/s1. NMR and LCMS data of *Pancratium* bulb extract. Figure S1: H-NMR of *Pancratium* bulb extract. Figure S2: HSQC-DEPT of *Pancratium* bulb extract. Figure S3: HMBC of *Pancratium* bulb extract. Figure S4: COSY of *Pancratium* bulb extract. Figure S5: TOCSY of *Pancratium* bulb extract. Figure S6: NOESY of *Pancratium* bulb extract. Table S1: Principal assignments of identified alkaloids in Bulb extract as obtained from NMR spectra. Figure S7: chromatogram related to specie at *m*/*z* 334. Figure S8: specie at *m*/*z* 334, RT 1.7 min. Figure S9: specie at *m*/*z* 334, RT 2.3 min. Figure S10: chromatogram related to specie at *m*/*z* 288. Figure S11: specie at *m*/*z* 288 lycorine RT 1,9 min. Figure S12: specie at *m*/*z* 288 epilycorine RT 2.45 min. Figure S13: specie at *m*/*z* 288 lycorine type RT 3.8 min. Figure S14: specie at *m*/*z* 288 lycorine type RT 3.9 min. Figure S15: chromatogram related to specie at *m*/*z* 302. Figure S16: specie at *m*/*z* 302 oduline RT 3.3 min. Figure S17: specie at *m*/*z* 302 RT 4.4 min. Figure S18: specie at *m*/*z* 302 RT 4.5 min. Figure S19: specie at *m*/*z* 302 RT 6.0 min lycorine derivatives. Figure S20: specie at *m*/*z* 302 RT 7.3 min. Figure S21: specie at *m*/*z* 302 RT 7.84 min. Figure S22: chromatogram related to specie at *m*/*z* 318. Figure S23: specie at *m*/*z* 318 crinamidine RT 5.6 min. Figure S24: chromatogram related to specie at *m*/*z* 306. Figure S25: specie at *m/z* 306 RT 5 min. Figure S26: specie at *m*/*z* 306 RT 7 min. Figure S27: chromatogram related to specie at *m/z* 494. Figure S28: specie at *m*/*z* 494 RT 4.5 min.
