# Supplementary material for: Chemical Characterization and Cytotoxic and Antioxidant Activity Evaluation of the Ethanol Extract from the Bulbs of Pancratium maritimun Collected in Sicily
Source: Molecules. 2023 May 9;28(10):3986. doi: 10.3390/molecules28103986 (PMC10222022; doi:10.3390/molecules28103986)
Supplement: Supplementary file 1 [file molecules-28-03986-s001.zip › molecules-2338491-supplementary.pdf]

Supplementary material

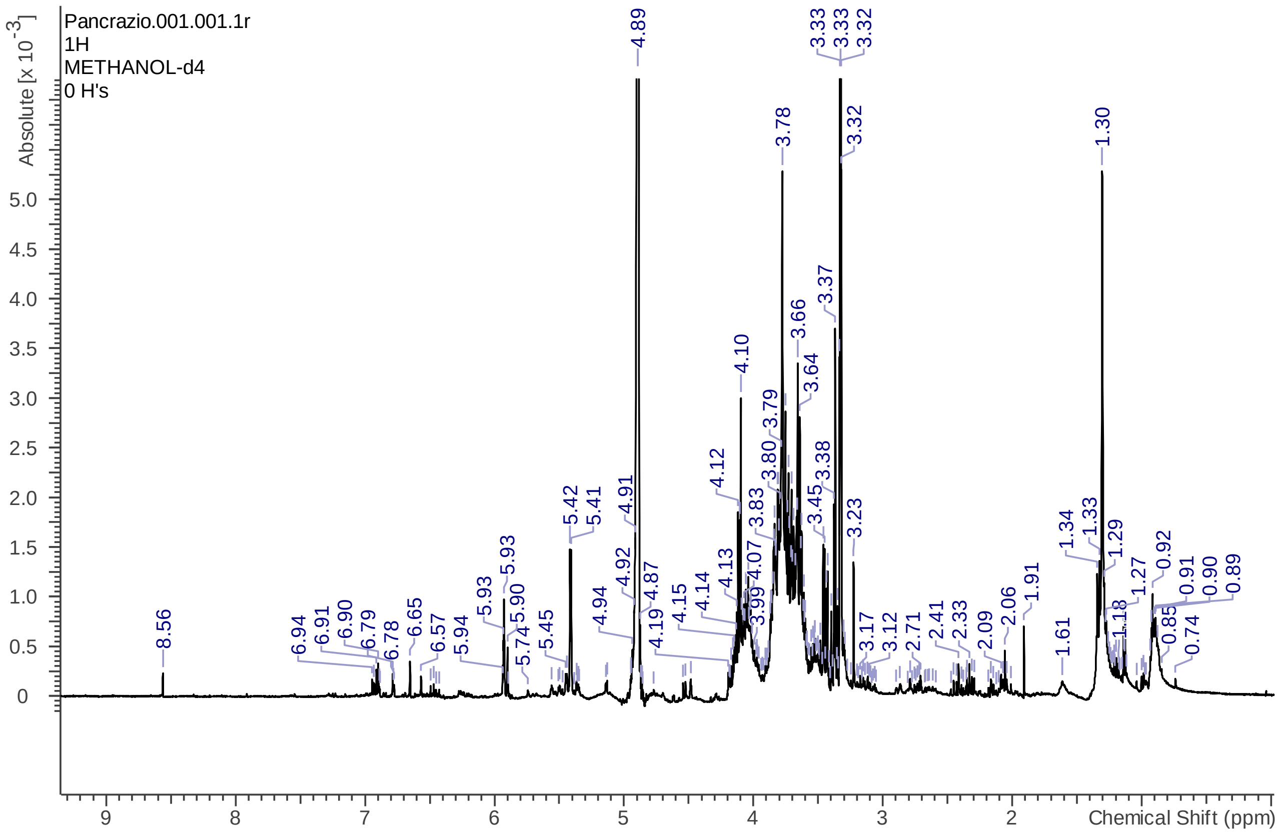

Figure S1: H-NMR of *Pancratium* bulb extract.

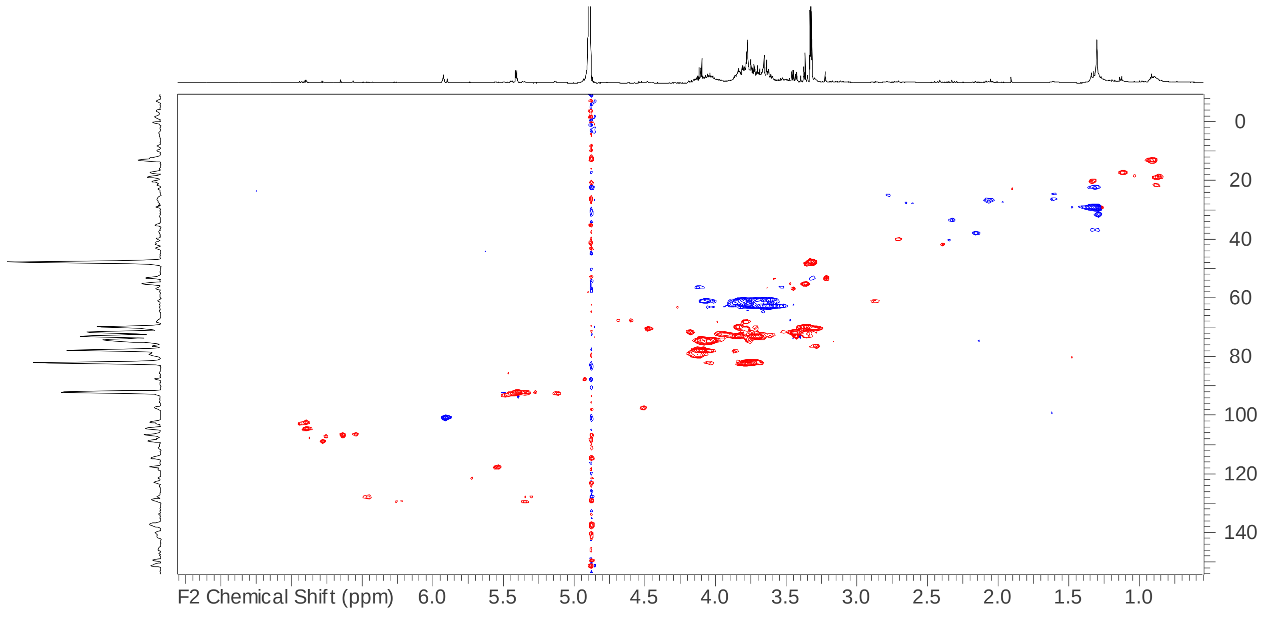

Figure S2: HSQC-DEPT of *Pancratium* bulb extract.

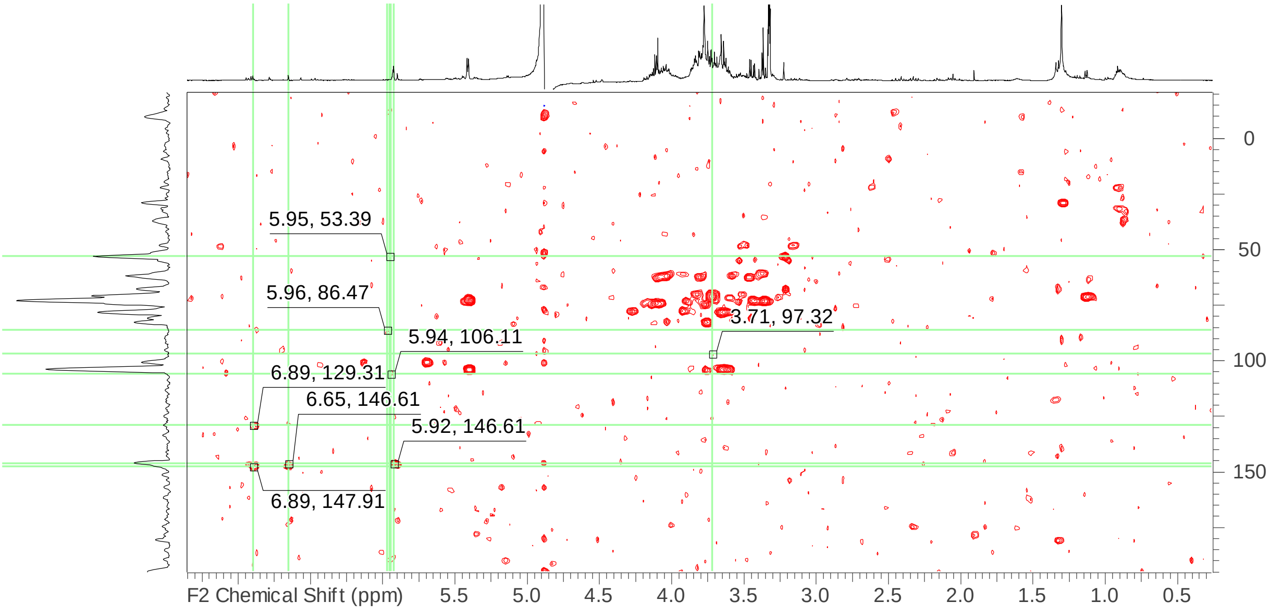

Figure S3: HMBC of *Pancratium* bulb extract.

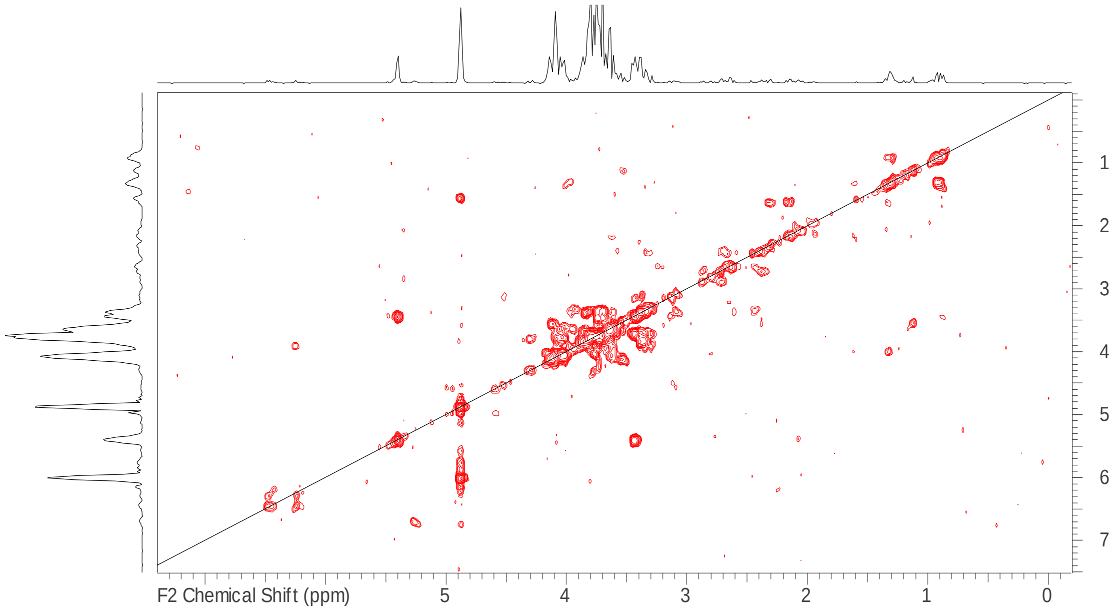

Figure S4: COSY of *Pancratium* bulb extract.

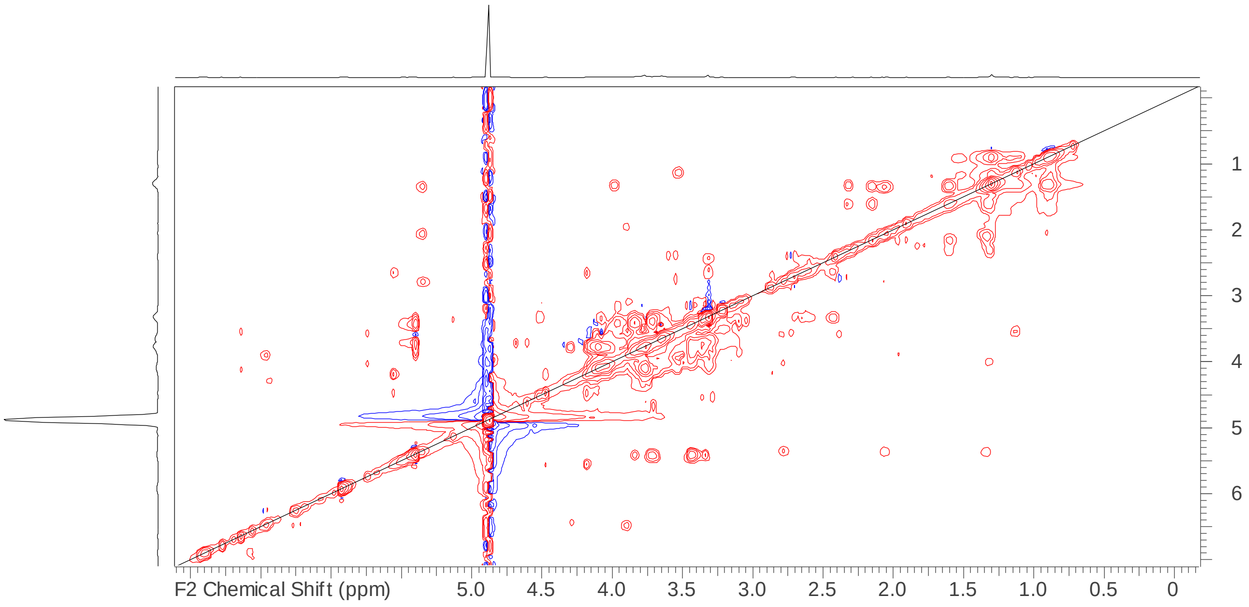

Figure S5: TOCSY of *Pancratium* bulb extract.

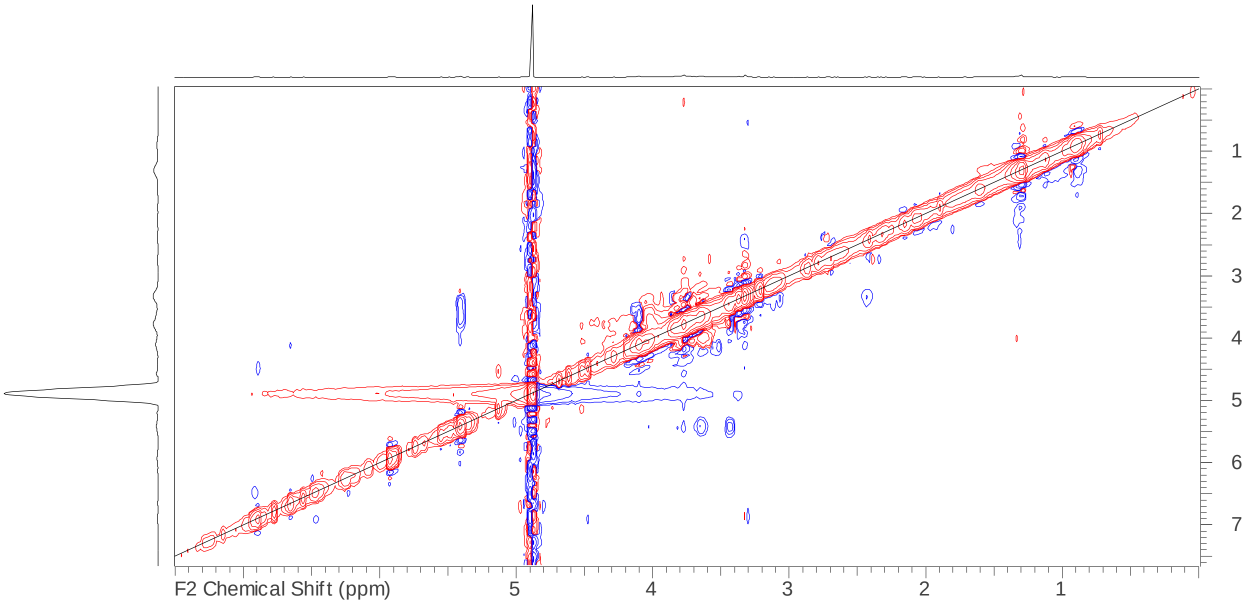

Figure S6: NOESY of *Pancratium* bulb extract.

Table S1: Principal assignments of identified alkaloids in Bulb extract as obtained from NMR spectra.

Lycorin type	F2 (ppm)	F1 (ppm)	HMBC	COSY/TOCSY	NOESY
1	4.57	70.51			6.98
2	4.27	71.78			
3	5.64	117.73			
4	2.42	33.72		3.32	
5	3.32	52.97		3.32	
7	3.46	55.45			
8	6.74	107.08			
11b	2.96	60.98			
11	6.98	104.85	147.9		4.57
12	6.02	101.07	147.9		
Crinamidine type					
1	3.77	56.2			6.56
2	3.26	53.3		4.46	
3	4.46	70.8		3.26	
6	4.19-3.53	56.4			
10	6.56	103.5			
12	3.31-2.43	53.1			
Oduline/Lycorenine type					
6	5.93	97.10		125.1	
N-CH3	2.30	44.3			
3	5.53	117.3		3.18	

LC-MS data chromatograms and MS fragmentation spectras

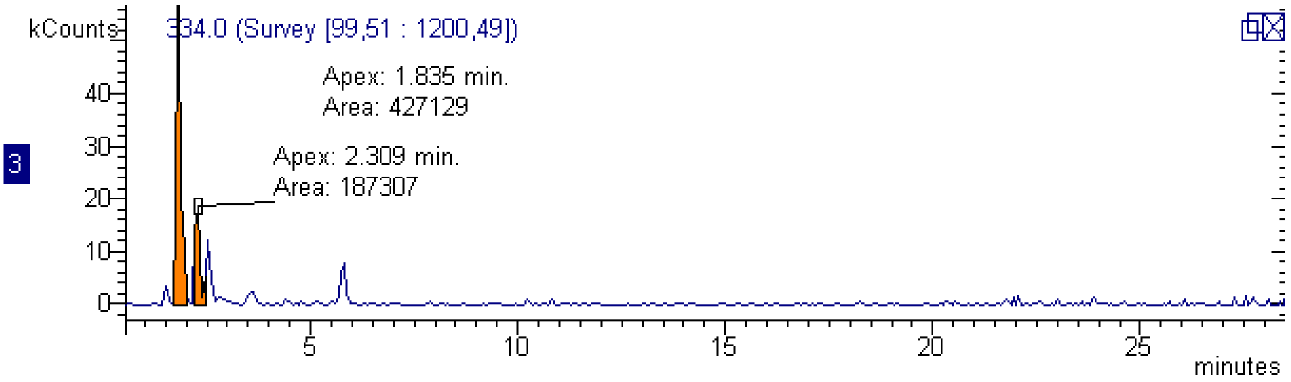

Figure S7: chromatogram related to specie at *m/z* 334.

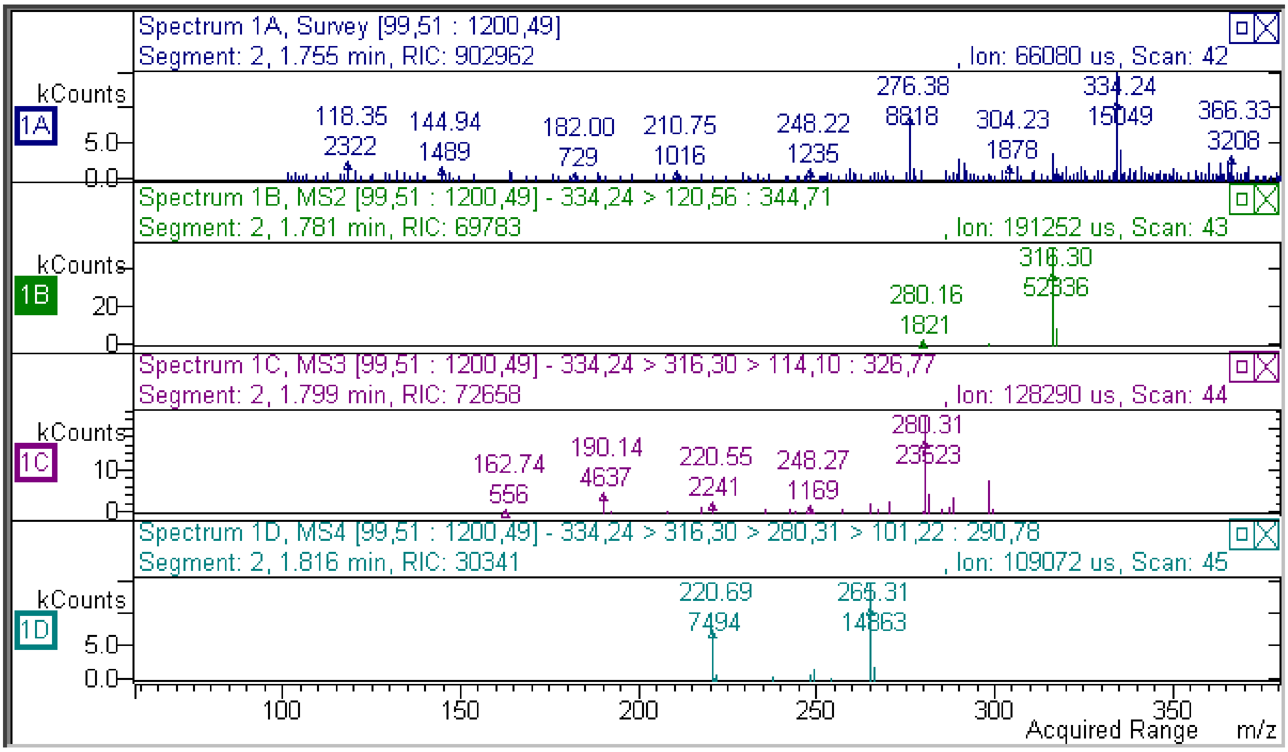

Figure S8: specie at *m/z* 334, RT 1.7 min.

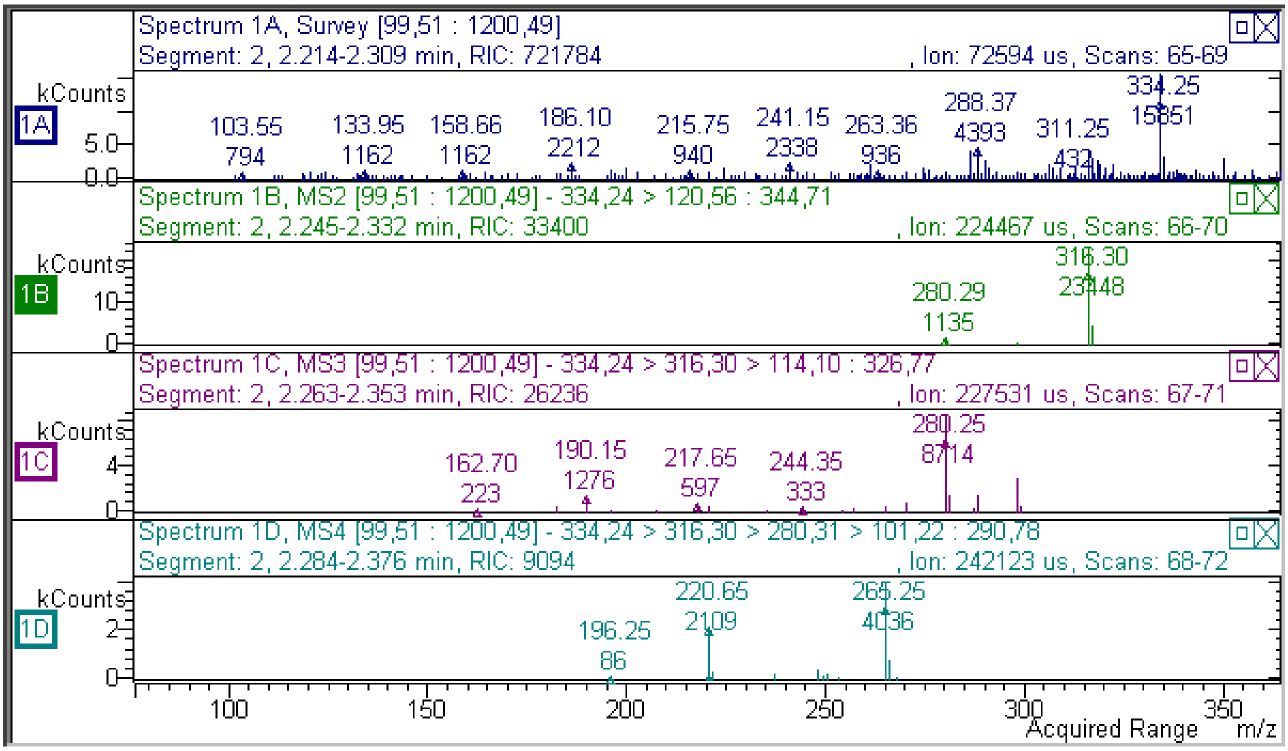

Figure S9: specie at *m/z* 334, RT 2,3 min.

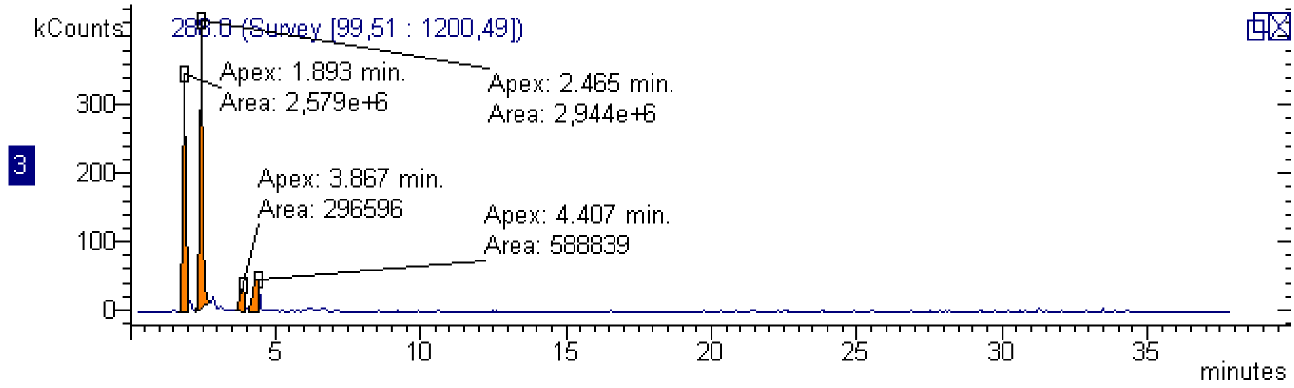

Figure S10: chromatogram related to specie at *m/z* 288.

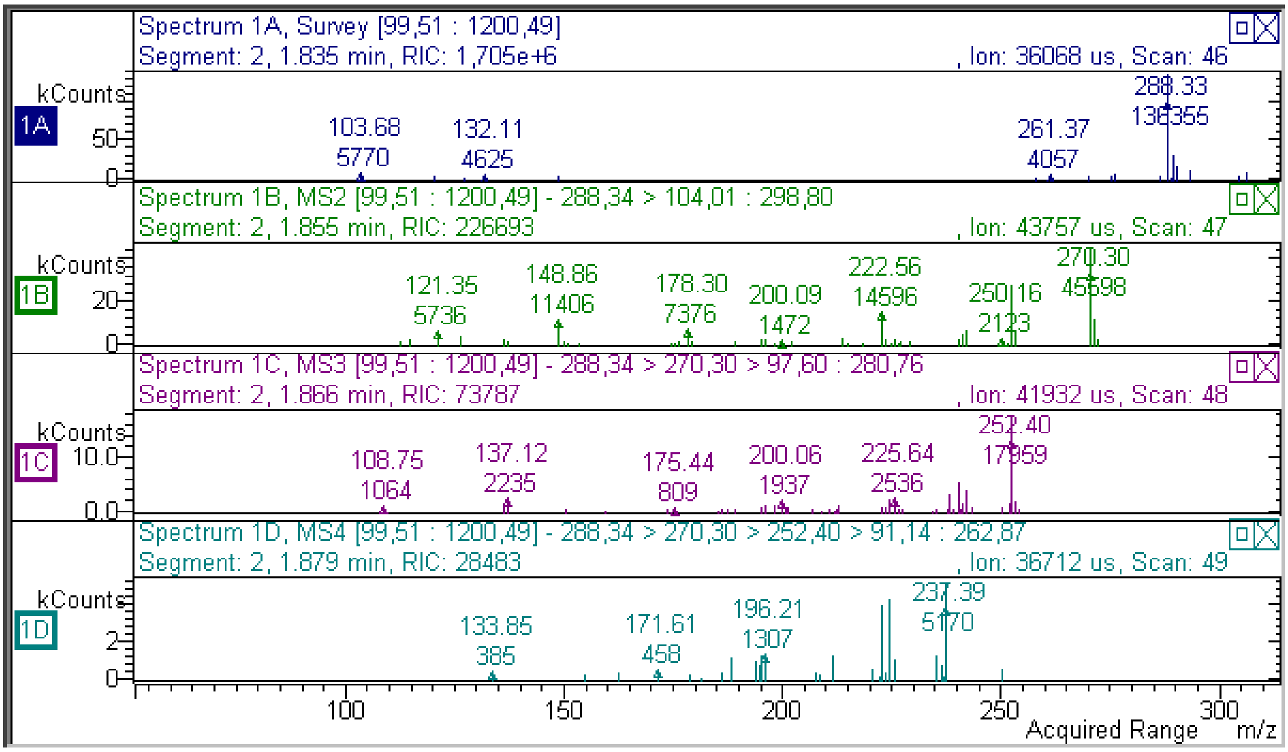

Figure S11: specie at *m/z* 288 lycorine RT 1,9 min.

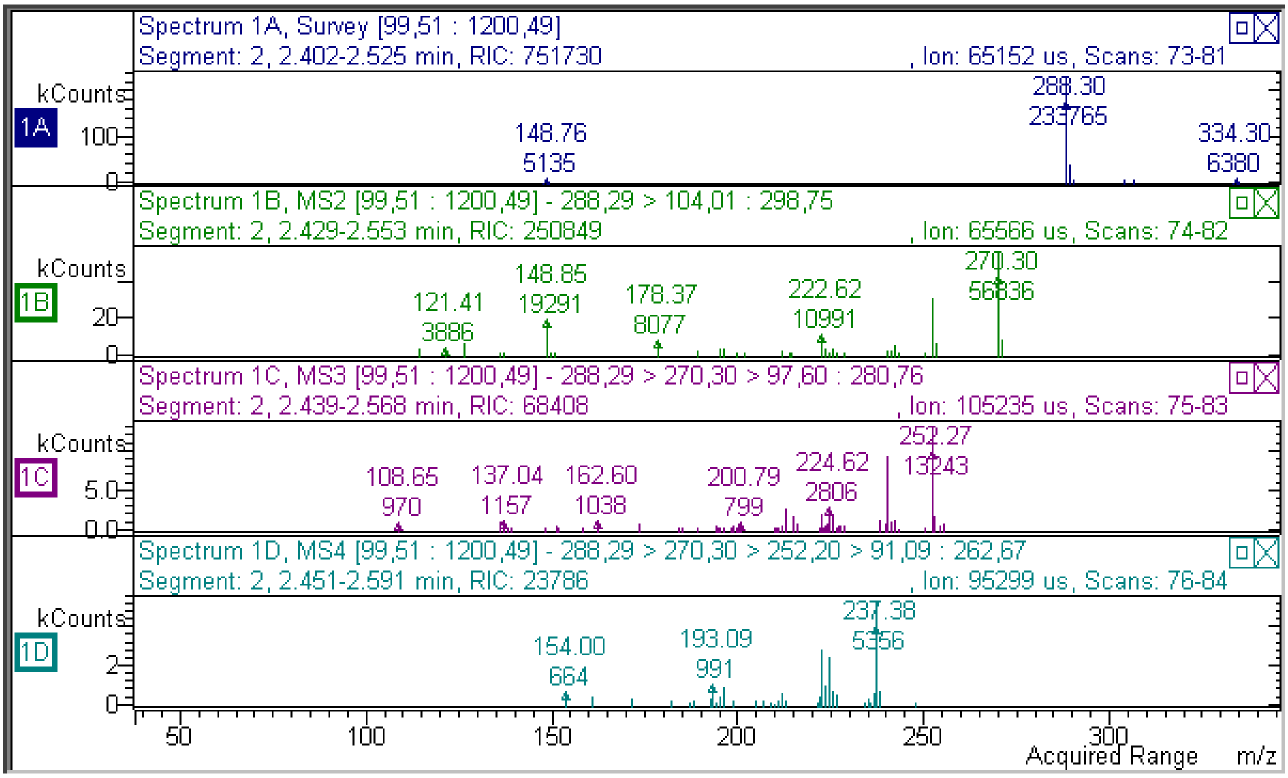

Figure S12: specie at *m/z* 288 epilycorine RT 2.45 min.

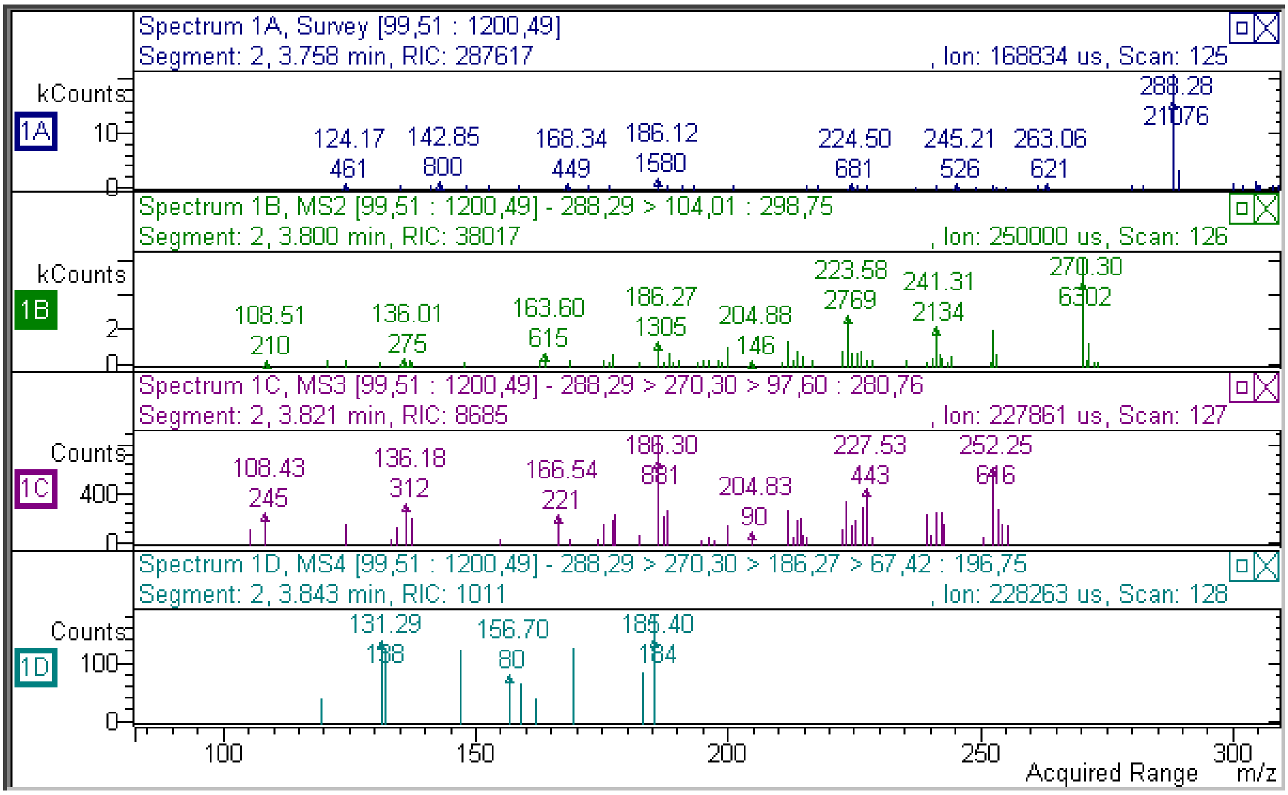

Figure S13: specie at *m/z* 288 lycorine type RT 3.8 min.

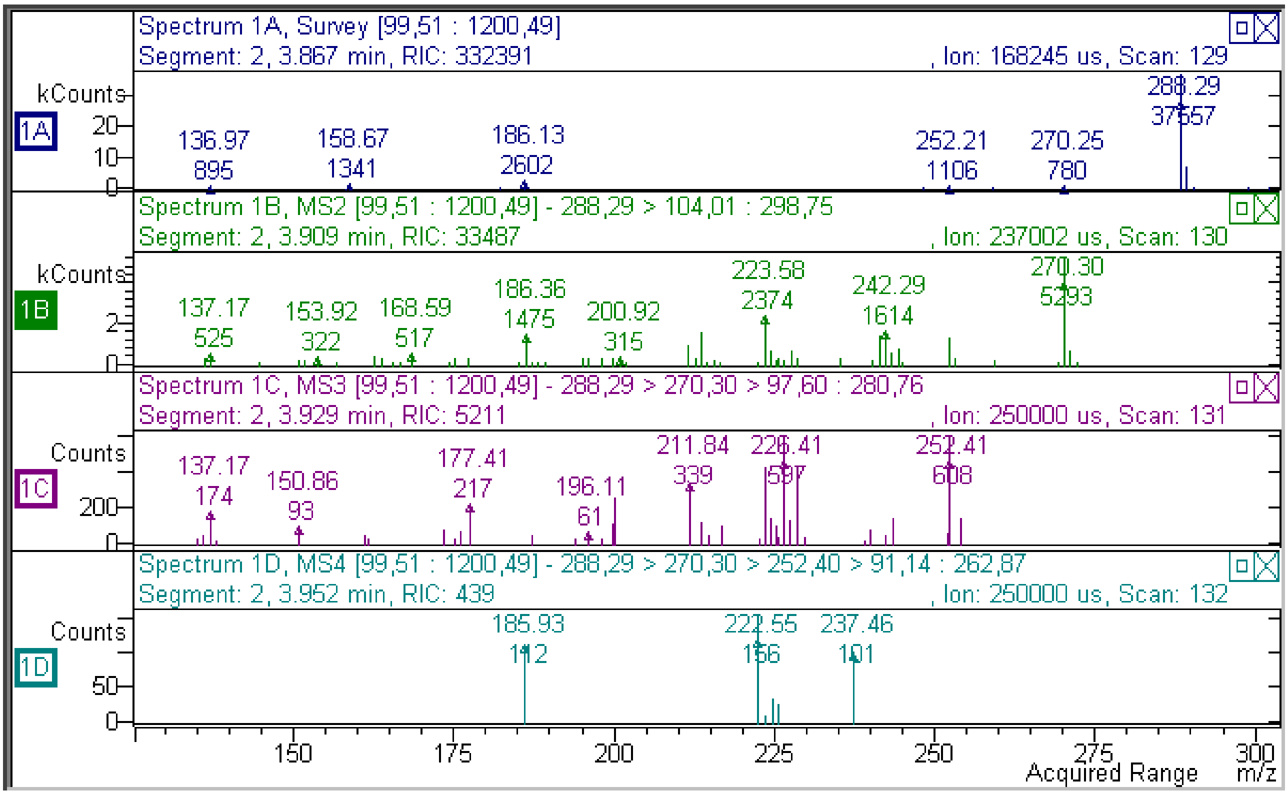

Figure S14: specie at *m/z* 288 lycorine type RT 3.9 min.

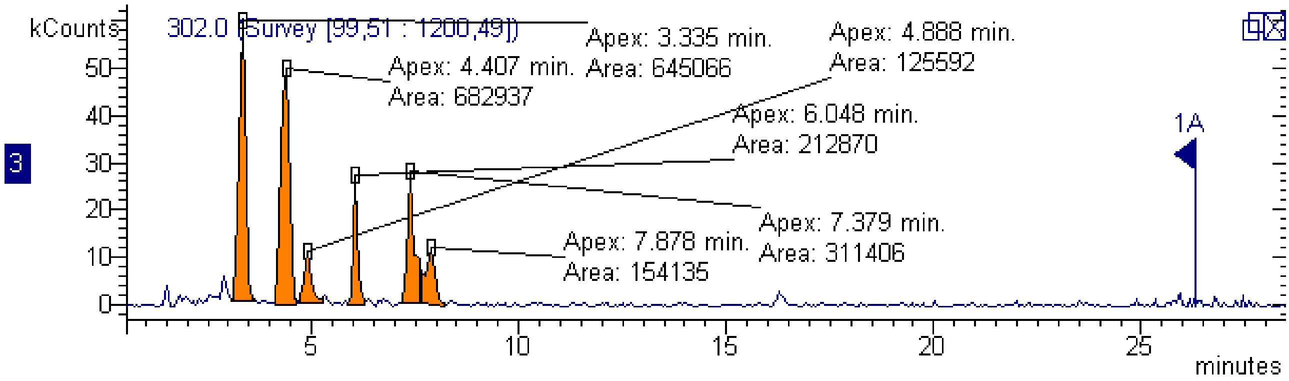

Figure S15: chromatogram related to specie at *m/z* 302.

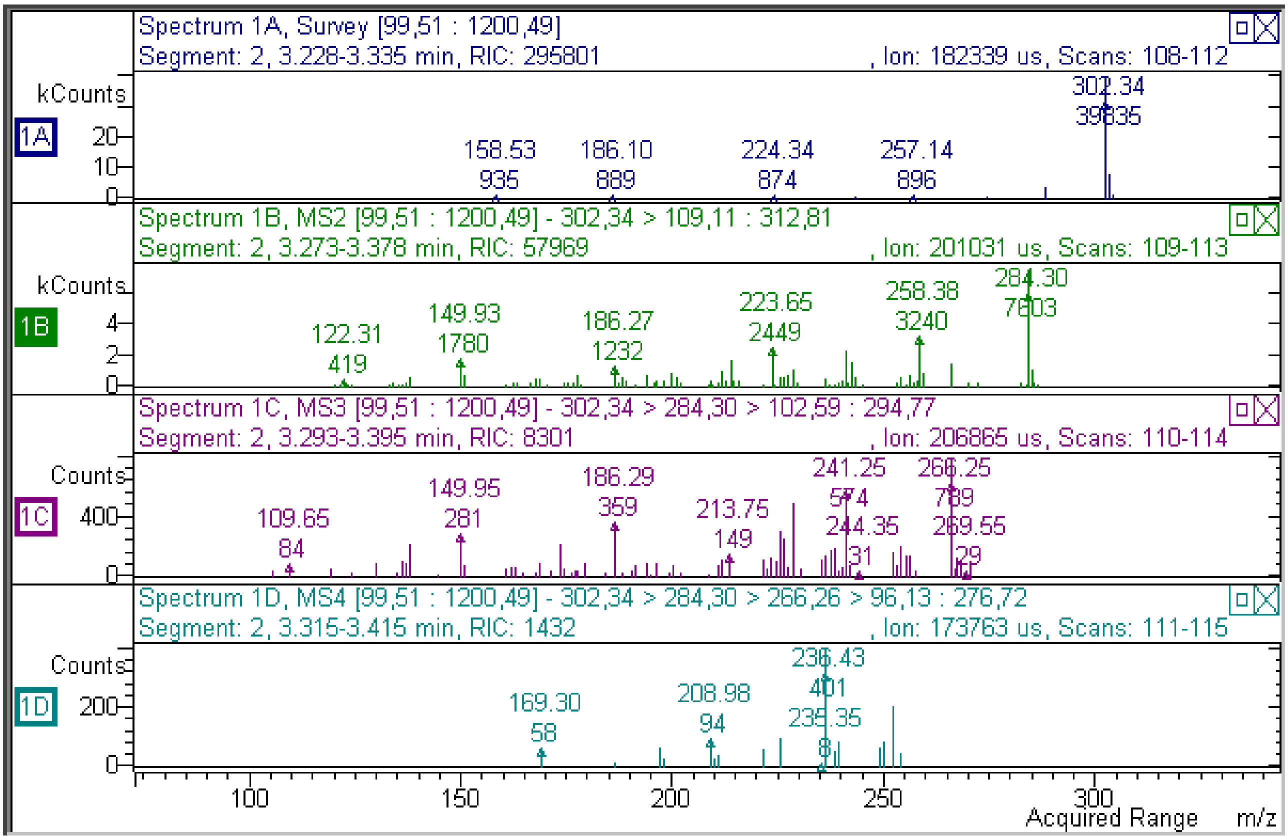

Figure S16: specie at *m*/*z* 302 oduline RT 3.3 min.

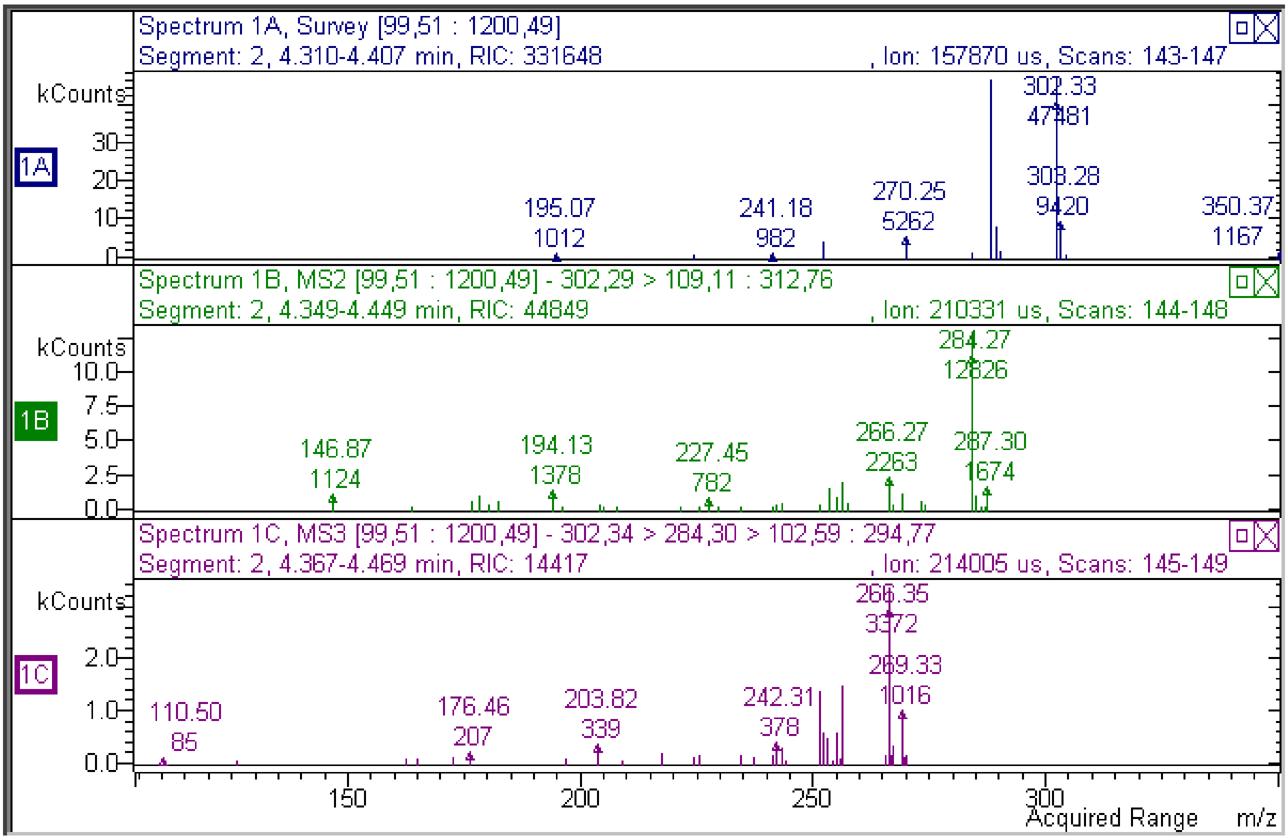

Figure S17: specie at *m*/*z* 302 RT 4.4 min.

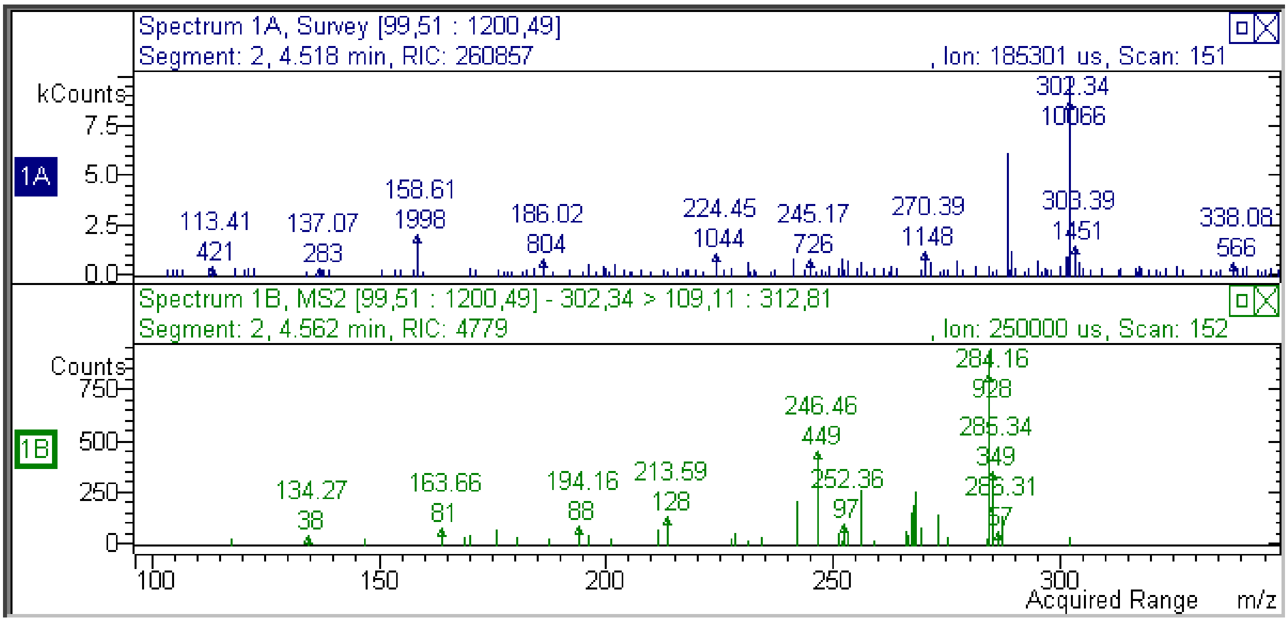

Figure S18: specie at *m/z* 302 RT 4.5 min.

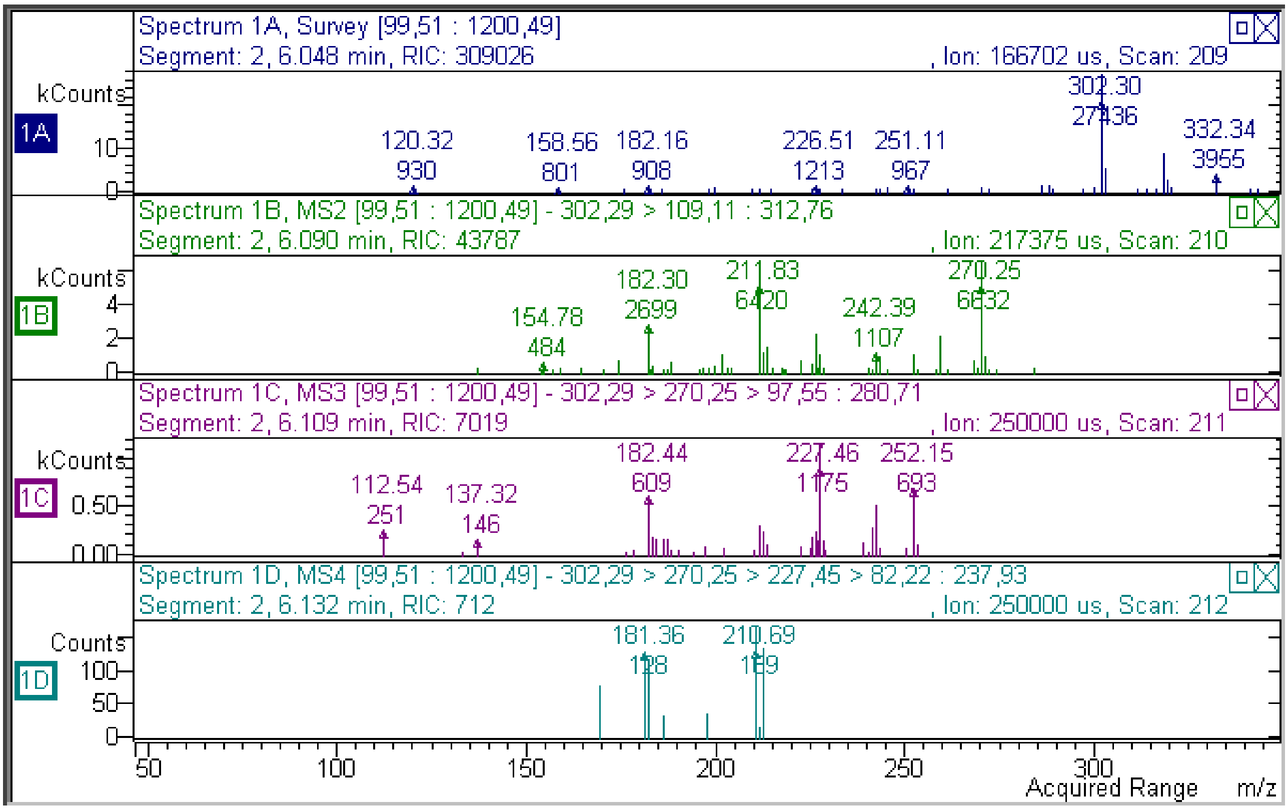

Figure S19: specie at *m/z* 302 RT 6.0 min lycorine derivatives.

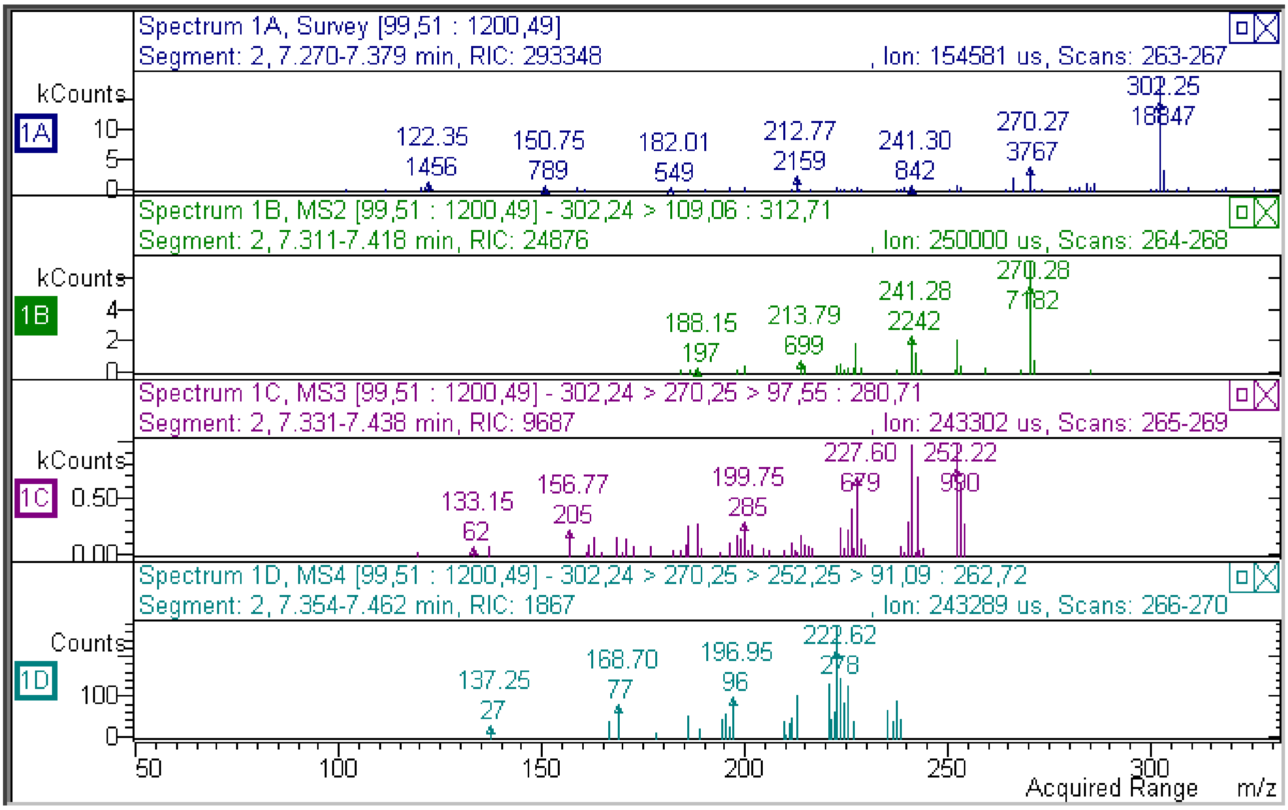

Figure S20: specie at *m/z* 302 RT 7.3 min.

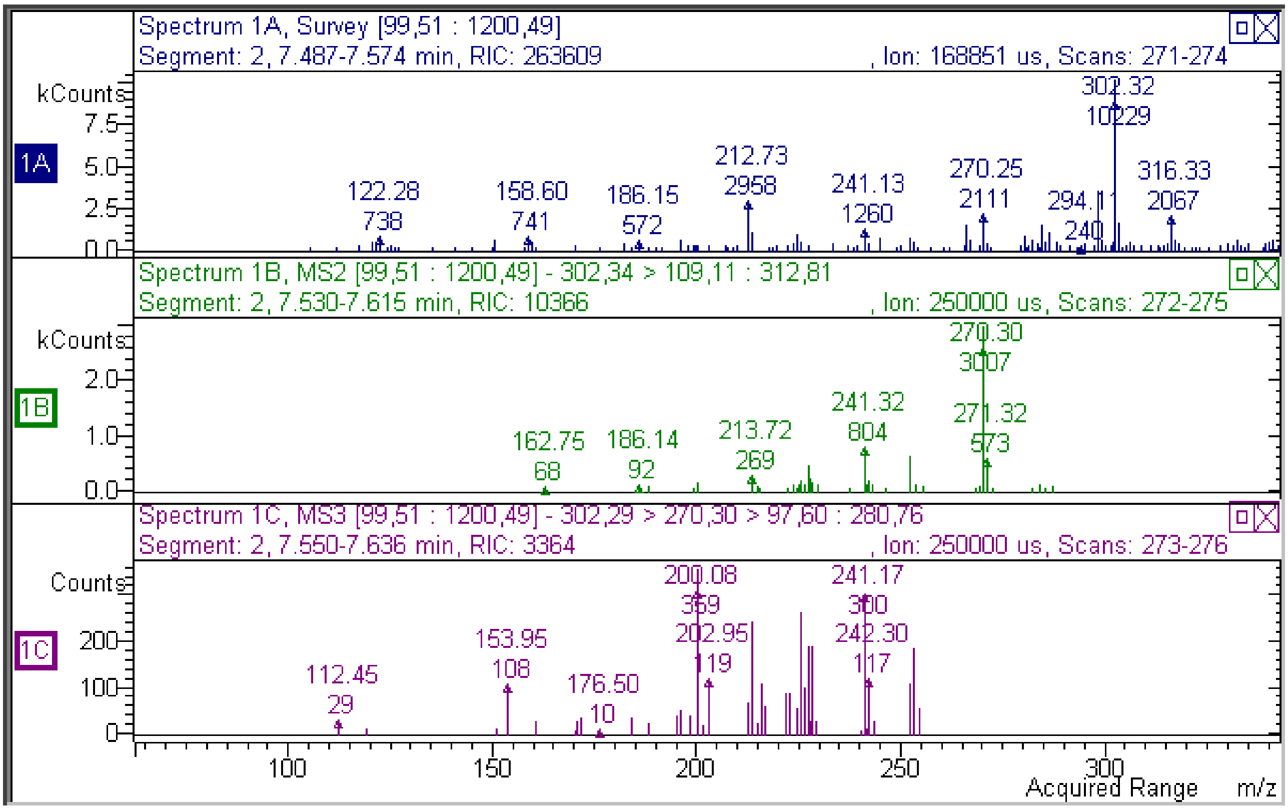

Figure S21: specie at *m/z* 302 RT 7.84 min.

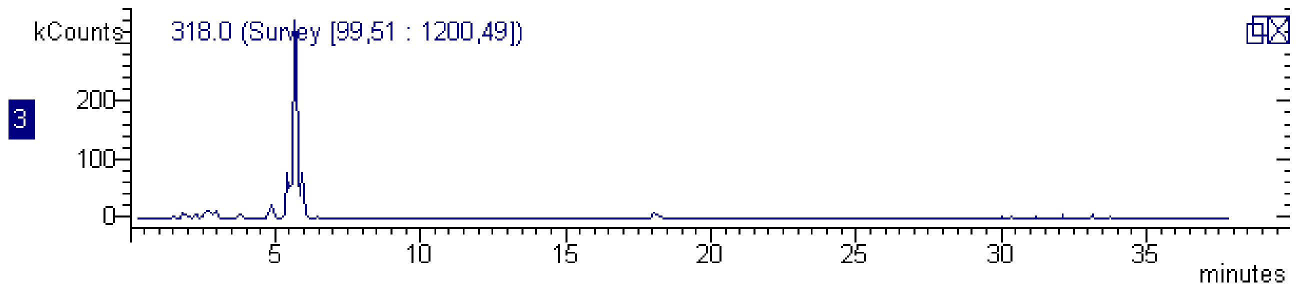

Figure S22: chromatogram related to specie at *m/z* 318.

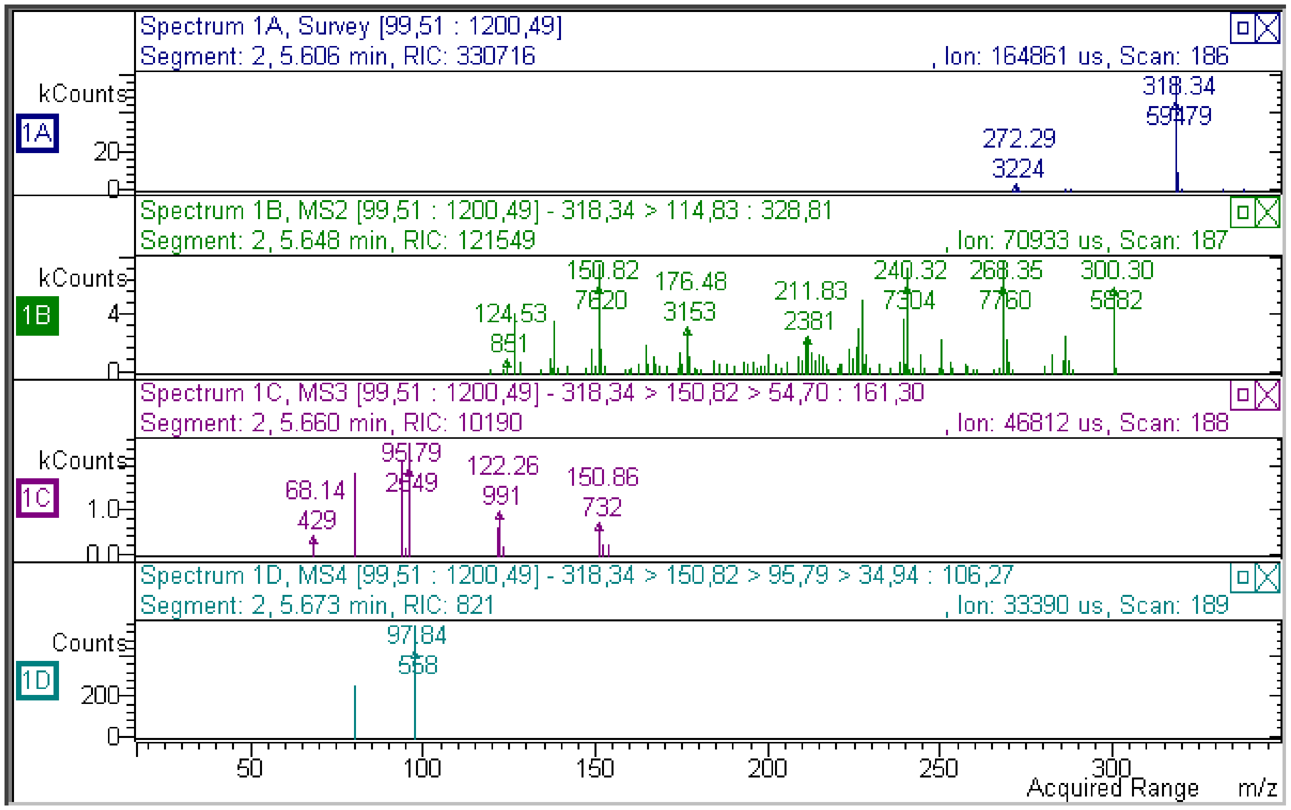

Figure S23: specie at *m/z* 318 crinamidine RT 5.6 min.

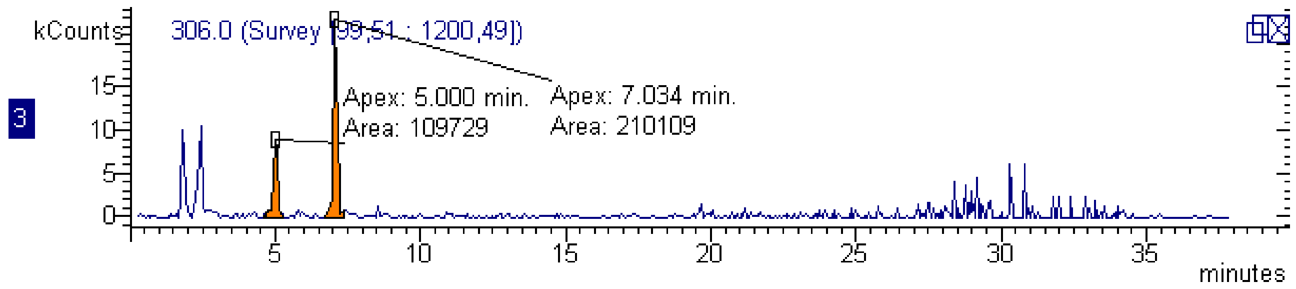

Figure S24: chromatogram related to specie at *m/z* 306.

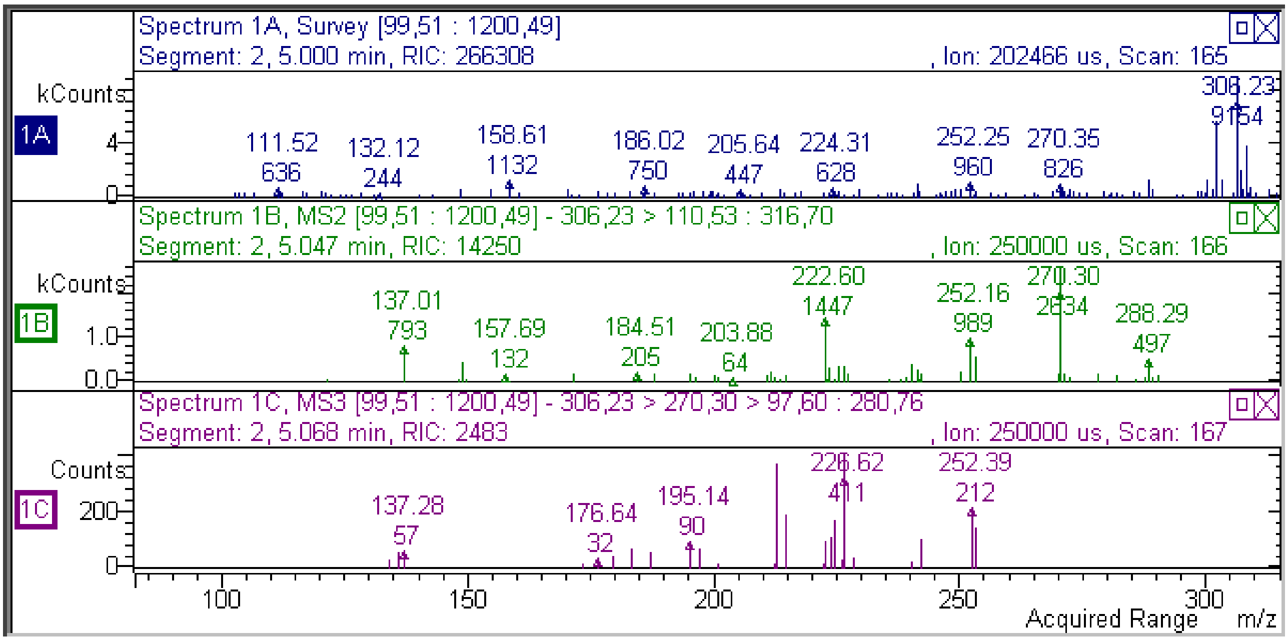

Figure S25: specie at *m/z* 306 RT 5 min.

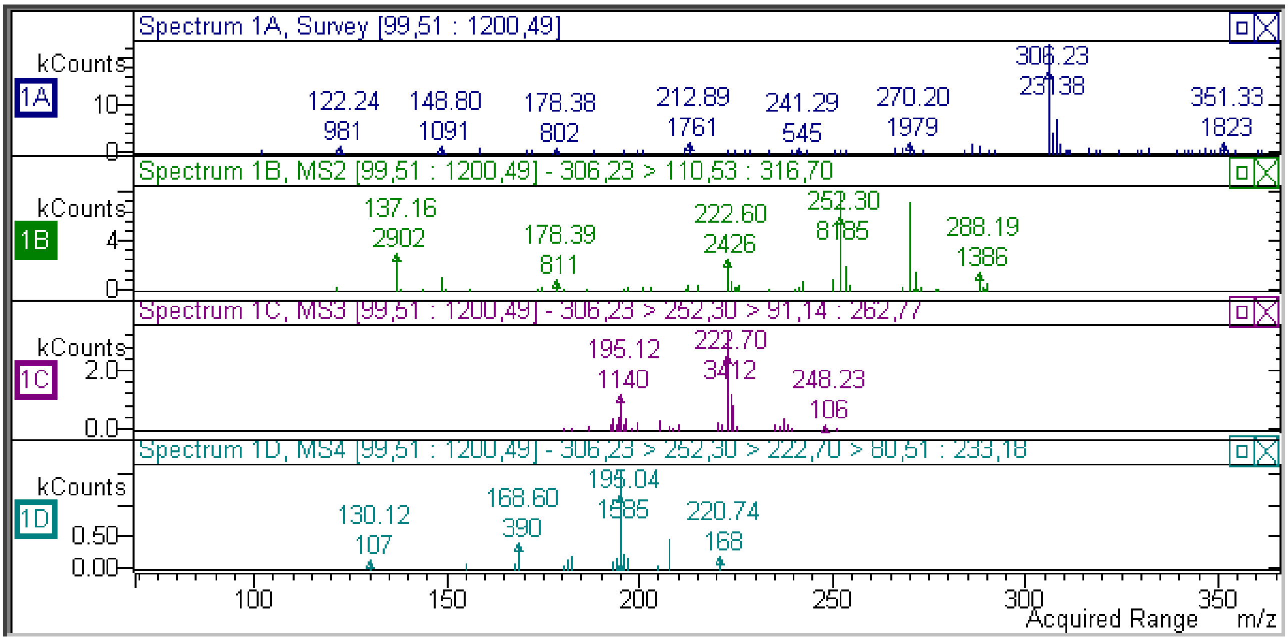

Figure S26: specie at *m/z* 306 RT 7 min.

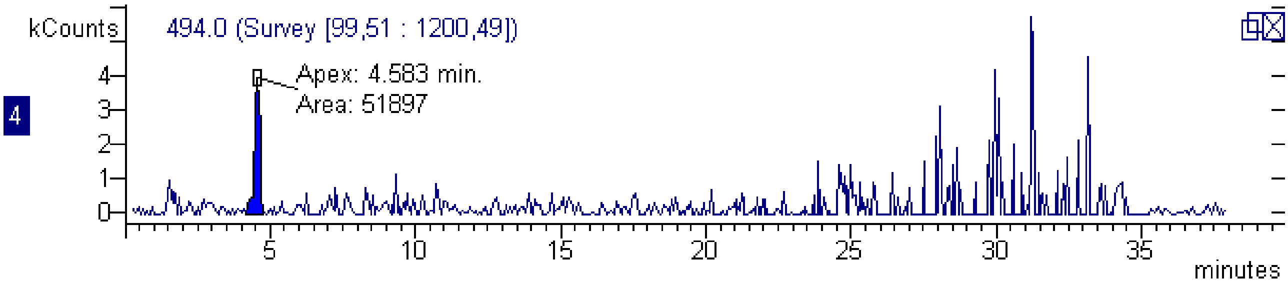

Figure S27: chromatogram related to specie at *m/z* 494.

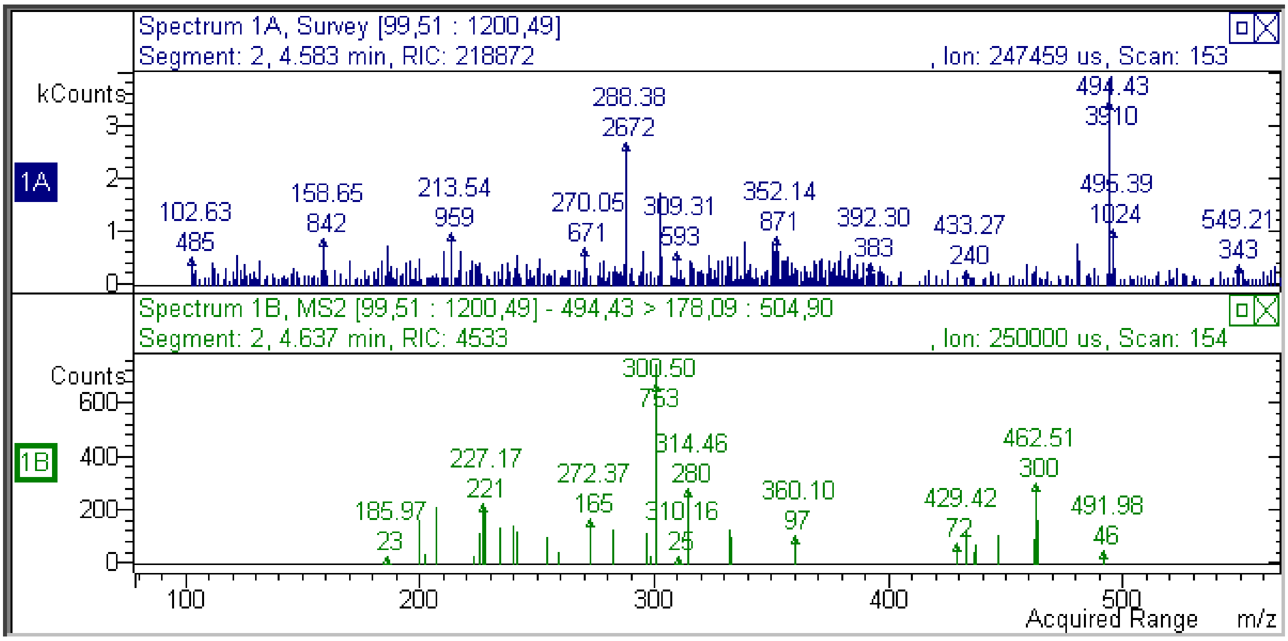

Figure S28: specie at *m/z* 494 RT 4.5 min.
